# Ethosuximide and Irritable Bowel Syndrome–Related Abdominal Pain: A Randomized Clinical Trial

**DOI:** 10.1001/jamanetworkopen.2025.51368

**Published:** 2026-01-08

**Authors:** Nicolas Kerckhove, Frank Zerbib, Marion Chambaz, François Mion, Alberto Zalar, Felix Goutorbe, Benoit Coffin, Laure Payen, Sabine Roman, Anne Guilngar, Bruno Pereira, Christian Dualé, Michel Dapoigny, Chloé Melchior, Julien Scanzi

**Affiliations:** 1 Université Clermont Auvergne, INSERM, UMR 1107 – NEURO-DOL, CIC 1405, CHU Clermont-Ferrand, Service de Gastroentérologie, Service de Pharmacologie Médicale, Direction de la Recherche Clinique et de l'Innovation, Clermont-Ferrand, France; 2 CHU de Bordeaux, Centre Médico-chirurgical Magellan, Hôpital Haut-Lévêque, Service de Gastroentérologie, Université de Bordeaux, INSERM CIC 1401, Bordeaux, France; 3 Université de Rennes 1, CHU Rennes, INSERM U1073, Service de Gastroentérologie, Rennes, France; 4 Hospices Civils de Lyon, Université de Lyon, Labtau U 13032 Inserm, Lyon, France; 5 Université de Rouen Normandie, INSERM, ADEN UMR1073, “Nutrition, Inflammation and microbiota-gut-brain axis”, CHU Rouen, CIC-CRB 1404, Service de Gastroentérologie, Service de Physiologie, Rouen, France; 6 CH Bayonne, Service de Gastroentérologie, Bayonne, France; 7 Assistance Publique- Hôpitaux de Paris, Service d’Hépato-Gastro-Entérologie, Hôpital Louis Mourier, DMU ESPRIT AP-HP Nord, Colombes, France; 8 Université Paris Cité, INSERM, Centre de recherche sur l’Inflammation, Paris, France; 9 CH Thiers, Service de Gastroentérologie, Thiers, France

## Abstract

**Importance:**

T-type calcium channels, particularly the Cav3.2 subtype, are involved in pain transduction. Experimental studies and human data suggested that increased T-type channels activity or expression contributes to visceral hypersensitivity in irritable bowel syndrome (IBS), and that their inhibition alleviates pain.

**Objective:**

To evaluate the therapeutic potential of ethosuximide, a T-type calcium channel blocker, for IBS-related abdominal pain.

**Design, Setting, and Participants:**

This proof-of-concept, multicenter, double-blinded, placebo-controlled randomized clinical trial started in February 2018 and completed in February 2022, with analyses performed between October 2023 and December 2024. Participants were adults meeting Rome IV criteria for IBS, treated in 10 gastroenterology departments in French university hospitals, with abdominal pain intensity rated at least 4 out of 10 during a 7-day run-in period.

**Interventions:**

Patients were randomized to receive ethosuximide or placebo daily for 12 weeks.

**Main Outcomes and Measures:**

The primary end point was the responder rate, defined as at least 30% reduction in mean abdominal pain intensity associated with a Subject Global Assessment of relief score at least 4 (ie, considerably or completely relieved). Secondary outcomes included safety, IBS symptom severity, and quality of life.

**Results:**

Of 161 enrolled patients, 124 were randomized (64 ethosuximide; 60 placebo). The mean (SD) age was 43.7 (14.9) years, 72 (58.1%) were women, the median (IQR) IBS duration was 5.0 (1.4-10.6) years, and the mean (SD) abdominal pain intensity was 6.0 (1.0). In the intent-to-treat analysis, responder rates did not differ significantly between groups (17 of 64 patients [26.6%] for ethosuximide vs 14 of 60 patients [23.3%] for placebo; relative risk, 1.14; 95% CI, 0.61-2.11). Ethosuximide was less well tolerated, with higher discontinuation rates (30 patients [46.9%] vs 13 patients [21.7%]; *P* = .003) and more adverse events (261 of 463 adverse events reported overall [56.4%] were determined to be caused by ethosuximide; *P* < .001), most commonly headaches, sleep disturbances, and nausea, compared with placebo.

**Conclusions and Relevance:**

In this randomized clinical trial, ethosuximide did not demonstrate efficacy over placebo for the treatment of IBS-related abdominal pain, and was associated with reduced tolerability. These findings do not support the use of ethosuximide for IBS pain management but highlight the need for development of more selective and better-tolerated T-type calcium channel modulators.

**Trial Registration:**

ClinicalTrials.gov Identifier: NCT02973542

## Key Points

**Question:**

What is the therapeutic efficacy of ethosuximide, a Cav3.2 blocker which has demonstrated analgesic activity in animal models of irritable bowel syndrome (IBS), in patients with IBS-related abdominal pain?

**Findings:**

In this randomized clinical trial in 124 patients assessing ethosuximide to improve IBS-related abdominal pain, intention-to-treat analysis showed no significant difference in responder rates between ethosuximide and placebo. Ethosuximide had limited tolerability, with higher discontinuation rates, and induced more adverse events compared with placebo.

**Meaning:**

These findings suggest ethosuximide is not recommended as treatment for IBS-related abdominal pain.

## Teaser

This randomized clinical trial examines the therapeutic potential of ethosuximide, a T-type calcium channel blocker, for irritable bowel syndrome–related abdominal pain.

## Introduction

Irritable bowel syndrome (IBS) is a disorder of gut-brain interaction characterized by recurrent abdominal pain and altered bowel habits.^1^ It affects at least 3.8% of the global population,^2,3^ substantially impairs quality of life, and imposes considerable health care costs.^4^ The limited availability of effective treatments makes IBS a major public health concern.

Altered intestinal barrier function is thought to play a central role in IBS pathophysiology by sustaining low-grade mucosal inflammation and visceral hypersensitivity.^5^ Although immune activation has been observed in IBS without the features of classic inflammation, the concept of inflammation in IBS remains debated.^6^ Visceral hypersensitivity, present in most patients with IBS,^7^ involves afferent sensitization and ion channel remodeling.^8^

T-type calcium channels, particularly the Cav3.2 subtype, are key modulators of pain.^9,10^ Their inhibition prevents visceral hypersensitivity in animals models.^11,12^ In humans, Cav3.2 channels are overexpressed in the colonic mucosa of patients with IBS.^13^ To our knowledge, only 1 nonrandomized, uncontrolled, open-label clinical trial has been conducted assessing a Cav3.2 channel blocker in this population, showing a potential benefit but providing a low level of evidence.^14^ We therefore conducted a multicenter, double-blind, placebo-controlled randomized clinical trial to rigorously assess the analgesic effect of ethosuximide in IBS-related abdominal pain.

## Methods

This randomized clinical trial was approved by the French medical ethics committee and by the French competent authorities. All participants provided written informed consent. Reporting followed the Consolidated Standards of Reporting Trials (CONSORT) reporting guideline. The protocol and statistical analysis plan have been published elsewhere^15^ and are available in Supplement 1.

There were a few deviations from the original statistical analysis plan. Genetic analysis of cytochrome P450 was not conducted for feasibility reasons. Recruitment difficulties led to the interim analysis being performed after 50 randomized patients instead of 100. Additionally, the target sample size was reduced following the results of the interim analysis.

### Study Design

The trial began in February 2018 and was completed in February 2022. Analyses were conducted between October 2023 and December 2024. This multicenter, double-blind, placebo-controlled randomized clinical trial assessed the efficacy and safety of ethosuximide in patients with IBS-related abdominal pain. Each patient participated for 16 weeks, including a 1-week run-in period, 12 weeks of treatment, and 3 weeks of follow-up. Randomization used computer-generated 6-block sequences stratified by center, with sequentially numbered containers.

### Study Objectives

The primary objective was to evaluate the efficacy of ethosuximide vs placebo on IBS-related abdominal pain using an 11-point numeric rating scale and the Subject Global Assessment of Relief (SGA).^16^ Secondary objectives included changes in IBS severity (assessed using the Irritable Bowel Severity Scoring System [IBS-SSS]^17^), and quality of life (assessed using Gastrointestinal Quality of Life Index [GIQLI]^18^ and EQ-5D-3L^19^). All questionnaires and scales were completed by patients, and their completion was verified at each visit.

### Inclusion and Exclusion Criteria

Eligible adults met Rome IV criteria,^20^ had abdominal pain for more than 3 months with a mean intensity of at least 4 out of 10 during the previous week,^21^ and were unresponsive to standard treatments. Exclusion criteria included diabetes, other dominant chronic pain conditions, severe depression, and epilepsy.

Major protocol deviations (screening failure, nonadherence, analgesic changes, or missing end points) led to exclusion from the modified intent-to-treat (mITT) population. To minimize bias, particular attention was paid to randomization procedures and group comparability.

### Investigational Medicinal Product

Patients received either ethosuximide or placebo in addition to their usual IBS treatments. No changes in concomitant regimens were allowed except for mild analgesics (eg, acetaminophen, nonsteroidal anti-inflammatory drugs).

#### Ethosuximide and Placebo

**Figure 1.**
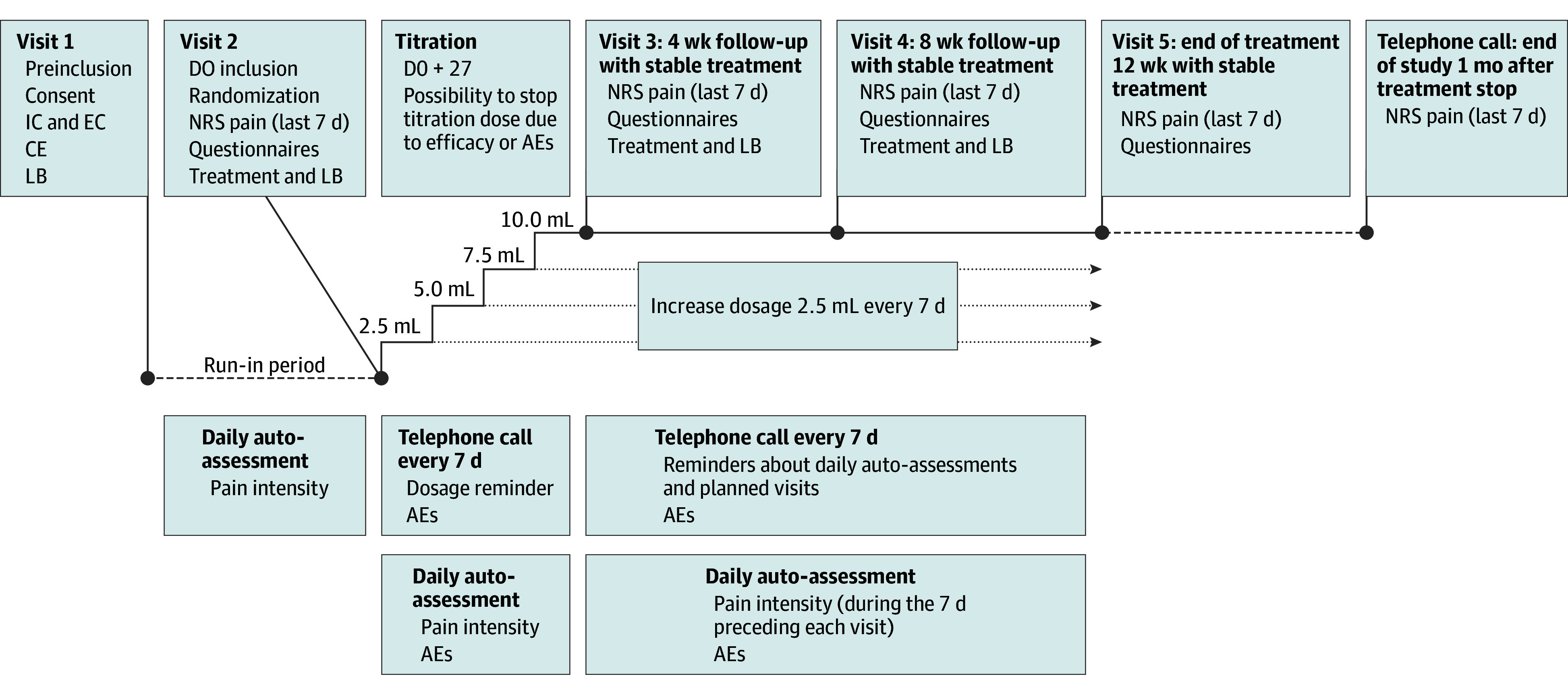
Study Design AE indicates adverse events; CE, clinic examination; DO, day 0; EC, exclusion criteria; IC, inclusion criteria; LB, logbook; NRS, Numeric Rating Scale.

Ethosuximide and placebo were administered daily for 12 weeks. The dose was gradually increased during a 28-day titration period to a maximum of 500 mg per day (Figure 1). Dose reduction was permitted if adverse events (AEs) occurred.

#### Blinding Conditions and Adherence Ascertaining

The placebo matched ethosuximide in flavor, sugar content, and packaging. The coordinating center’s pharmacy prepared and labeled the bottles with specific 4-digit codes and shipped them to the participating centers. Only local pharmacists managed dispensing and monitored adherence by verifying of the volume of syrup used relative to theoretical dosages. The statistician (B.P.) remained blinded throughout all analyses.

### Primary End Point

The primary composite end point was the responder rate, defined as a reduction of at least 30% in mean weekly abdominal pain intensity from baseline and an SGA score of at least 4 our of 5 (ie, considerably relieved or completely relieved). Unlike the European Medicines Agency guidelines,^22^ a 4 out of 5 SGA threshold was considered clinically relevant, and assessments were performed monthly to reduce patient burden.

### Clinical Interventions

#### Enrollment

Patients being treated for IBS were preselected during consultations and invited to participate across 10 French academic gastroenterology departments. At visit 1, patients received a diary and instructions for recording daily abdominal pain and AEs over 7 days to establish baseline values. At visit 2, mean abdominal pain scores from the previous week were collected; a mean score of at least 4 was required for randomization. Patients completed questionnaires and received medication for the first 4-week treatment period. At each monthly visit, patients completed questionnaires and the SGA scale. Medication for the next treatment period was dispensed. Three weeks after treatment completion, a follow-up phone call was made to collect residual AEs.

#### Safety

AEs were collected through patient diaries and scheduled phone calls, categorized by severity and relatedness. Given to the long half-life of ethosuximide (60 hours), an additional phone call within 3 weeks after discontinuation was performed to capture delayed AEs.

### Statistical Analysis

The sample size was initially estimated at 130 randomized patients per group to provide 90% power to detect a 20% absolute difference in responder rate (2-sided α = .05), assuming responder rates of 35% for placebo and 55% for ethosuximide.^23^ A blinded interim analysis was initially planned after 100 patients, according to the Lan and DeMets α spending function (East software version 6.2; Cytel), with a type I error of .025. Due to recruitment challenges, the interim analysis was performed after 50 randomized patients. The analysis, reviewed by 2 independent experts, revealed no major safety concerns and responder rates of 14% and 36% in the ethosuximide and placebo groups. Based on these results, the required sample size was recalculated^24,25^ to 128 randomized patients (64 per group), assuming 15% and 40% responder rates in the placebo and ethosuximide groups, respectively (α = .025; power = 84%). To account for potential nonrandomization, up to 160 patients were to be enrolled.

Primary analyses were performed in ITT, mITT, and per-protocol (PP) populations. Results are expressed as relative risks (RRs) with 95% CIs. The CIs for binary percentages were obtained with binomial exact method. Patients who were not adherent to treatment or discontinued treatment were considered nonresponders. Responder rates were compared using χ^2^ tests, and a prespecified multivariate model using a generalized linear mixed model (Poisson regression with a log link and robust variance^26^) accounted for fixed covariates (eg, sex, IBS subtype) and center-level variability. Modified Poisson regression was used with robust error variance to analyze binary outcomes. This approach directly estimates RRs rather than odds ratios, which are often less intuitive and may overestimate the effect when the outcome is common (>10%-15%).^27,28^ Compared with logistic regression, which yields odds ratios, the modified Poisson model provides a more clinically interpretable measure of association (RRs) and avoids the difficulty of communicating odds ratios to nonstatistical audiences.^29^ Compared with the log-binomial model, which also estimates RRs, the modified Poisson regression is preferred in practice because log-binomial models often face convergence problems and boundary issues, especially when probabilities are close to 0 or 1. The modified Poisson method is more stable computationally, while still yielding consistent estimates of RRs with correct inference when robust variance estimation is applied. For the analysis of the primary outcome, the intraclass correlation coefficient related to center effect was close to 0% and the *P* value for variance components of random effects at greater than .99. Primary end point components were adjusted for multiple testing using the Hochberg procedure.^30^

Continuous outcomes were analyzed using linear mixed models incorporating patient-level random effects (slope and intercept) nested within center-level random effects. Results were expressed as standardized mean differences (SMDs) with 95% CIs based on time-by-group interactions. The normality of residuals was assessed. Safety analyses used χ^2^ or Fisher exact tests.

Missing data were handled using multiple imputation by chained equations (MICE). The number of patients without missing data are specified in tables and legends. All analyses were performed using Stata software version 15.0 (StataCorp), with 2-sided *P* < .05 as significant. CI widths were not adjusted for multiplicity and should not be interpreted as formal hypothesis testing except for the primary end point and its components. Analyses were performed using Stata software version 15.0 (StataCorp).

## Results

A total of 161 patients with IBS were included, and 124 patients (mean [SD] age, 43.7 [14.9] years; 72 [58.1%] women) were randomized (64 to the ethosuximide group and 60 to the placebo group) between 2018 and 2022. Although the initial target of 128 randomized patients was not reached, the sample provided 82% statistical power. Patients who were not randomized did not meet the inclusion criterion of mean abdominal pain intensity of at least 4 out of 10 during the 7-day run-in period.

The study population had a median (IQR) IBS duration of 5.0 years (1.4-10.6) and a mean (SD) pain intensity of 6.0 (1.0). IBS subtypes were distributed as follows: 47 patients (37.9%) with IBS-C , 36 patients (29.0%) with IBS-D, and 39 patients (31.5%) with IBS-M. Baseline characteristics did not differ significantly between the study groups in the ITT, mITT, or PP populations (Table 1).

**Table 1.** Demographics and Baseline Characteristics of the Treatment Groups

Characteristics	Participants, No. (%)					
	ITT (n = 124)		Modified ITT (n = 99)		Per protocol (n = 69)	
	Ethosuximide (n = 64)	Placebo (n = 60)	Ethosuximide (n = 46)	Placebo (n = 53)	Ethosuximide (n = 28)	Placebo (n = 41)
Age, mean (SD), y	42.9 (15)	44.6 (15)	41.5 (14.6)	44.1 (14.9)	40.5 (14.9)	43.8 (14.0)
Sex						
Female	36 (56.2)	36 (60.0)	28 (60.9)	31 (58.5)	15 (53.6)	23 (56.1)
Male	28 (43.8)	23 (38.3)	18 (39.1)	22 (41.5)	12 (46.4)	18 (43.9)
IBS type						
C	24 (37.9)	23 (38.3)	17 (37.0)	22 (41.5)	9 (32.1)	16 (39.0)
D	22 (34.4)	14 (23.3)	16 (34.8)	12 (22.6)	12 (42.9)	11 (26.8)
M	17 (26.6)	22 (36.7)	12 (26.0)	18 (34.0)	6 (21.4)	13 (31.7)
Unknown	1 (1.6)	1 (1.7)	1 (2.2)	1 (1.9)	1 (3.6)	1 (2.5)
IBS duration, median (IQR), y	5.2 (1.7-11.1)	4.4 (1.4-9.9)	7.4 (4.8-9.0)	4.3 (2.1-6.5)	7.8 (3.9-11.7)	3.3 (0.8-5.8)
Abdominal pain intensity, mean (SD)^a^	6.1 (1.2)	6.0 (1.5)	6.1 (1.2)	6.1 (1.4)	5.7 (1.2)	5.9 (1.1)
Analgesic and IBS treatment						
Antacids medications	8 (12.5)	10 (16.7)	8 (17.4)	3 (5.7)	7 (25.0)	3 (7.3)
Anticonvulsants	2 (3.1)	0	2 (4.3)	0	2 (7.1)	0
Anti-inflammatory	2 (3.1)	1 (1.7)	1 (2.2)	1 (1.9)	1 (3.6)	1 (2.4)
Antispasmodics	24 (37.5)	21 (35.0)	16 (34.8)	21 (39.6)	12 (42.9)	20 (48.8)
Intestinal transit regulators	26 (40.6)	22 (36.7)	18 (39.1)	19 (35.8)	12 (42.9)	15 (36.6)
Antidepressants	3 (4.7)	1 (1.7)	3 (6.5)	0	2 (7.1)	0
Weak analgesics (eg, paracetamol, nefopam)	11 (17.2)	8 (13.3)	7 (15.2)	9 (17.0)	6 (21.4)	6 (14.6)
Nutritional supplements or probiotics	5 (7.8)	7 (11.7)	5 (10.9)	6 (11.3)	3 (10.7)	4 (9.8)
Opioids	3 (4.7)	5 (8.3)	2 (4.3)	3 (5.7)	2 (7.1)	3 (7.3)
Others	6 (9.4)	4 (6.7)	5 (10.9)	3 (5.7)	1 (3.6)	2 (4.9)
Comorbidities						
Cardiovascular	7 (10.9)	8 (13.3)	6 (13.0)	5 (9.4)	2 (7.1)	3 (7.3)
Neuropsychiatric	12 (18.7)	7 (11.7)	10 (21.7)	5 (9.4)	8 (28.6)	4 (9.8)
Respiratory	5 (8.3)	2 (3.3)	3 (6.5)	0	1 (3.6)	0
Digestive tract	18 (28.1)	17 (28.3)	13 (28.3)	13 (24.5)	9 (32.1)	11 (26.8)
Urogenital	12 (18.7)	13 (21.7)	8 (17.4)	7 (13.2)	6 (21.4)	7 (17.1)
Liver	2 (3.1)	1 (1.7)	1 (2.2)	1 (1.9)	0 (0.0)	1 (2.4)
Ear, nose, and throat	1 (1.6)	3 (5.0)	1 (2.2)	3 (5.7)	1 (3.6)	3 (7.3)
Dermatologic	2 (1.6)	5 (8.3)	1 (2.2)	3 (5.7)	1 (3.6)	3 (7.3)
Ocular	0	2 (3.3)	0	2 (3.8)	0	2 (4.9)
Immune	0	0	0	0	0	0
Endocrine and hematologic	2 (3.1)	2 (3.3)	1 (2.2)	1 (1.9)	0	1 (2.4)
Allergy	7 (10.9)	5 (8.3)	7 (15.2)	4 (7.5)	4 (14.3)	4 (9.8)
Rheumatologic	9 (14.1)	8 (13.3)	7 (15.2)	5 (9.4)	3 (10.7)	3 (7.3)

Abbreviations: IBS, irritable bowel syndrome; ITT, intention-to-treat;

^a^ Range, 1 to 10; higher score indicates greater pain.

**Figure 2.**
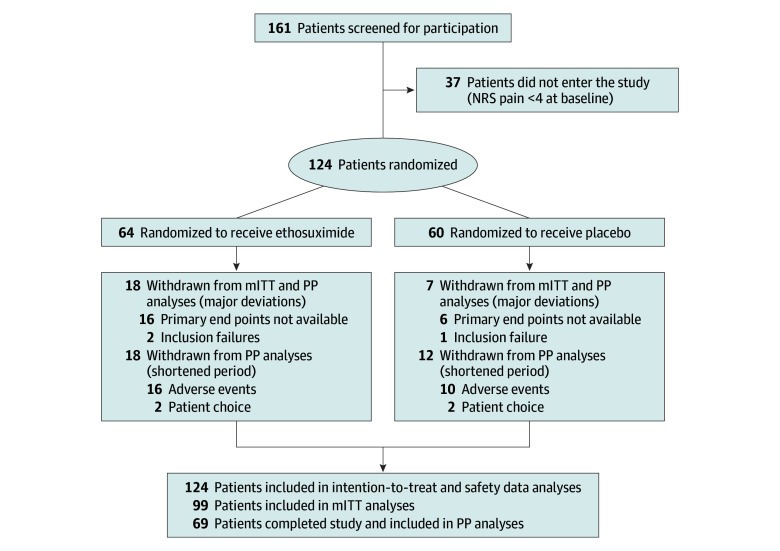
Enrollment Flowchart and Randomization of the Patients ITT indicates intention-to-treat; mITT, modified ITT; NRS, Numeric Rating Scale; PP, per protocol.

In the ethosuximide and placebo groups, 18 patients (28.1%) and 7 patients (11.7%), respectively, were excluded from the mITT population owing to major protocol deviations (16 vs 6 patients) or not meeting inclusion criteria (2 vs 1 patients). Among randomized participants, 28 patients (43.8%) in the ethosuximide group and 41 patients (68.3%) in the placebo group completed treatment (Figure 2).

### Primary Analysis

In the ITT population, no statistically significant difference was observed in responder rates between groups: 17 patients (26.6%; 95% CI, 15.7%-37.4%) in the ethosuximide group and 14 patients (23.3% [95% CI, 12.6%-34.0%]) in the placebo group met the criteria for response (RR, 1.73; 95% CI, 0.86-3.48) (Table 2). The mITT analysis yielded results consistent with the ITT findings. In contrast, the PP analysis demonstrated a statistically significant difference, with responder rates of 15 of 28 patients (53.6%; 95% CI, 35.1%-72.0%) in the ethosuximide group and 10 of 41 patients (24.4%; 95% CI, 11.2%-37.5%) in the placebo group (RR, 2.20; 95% CI, 1.15-4.18; χ^2^ *P* = .01) (Table 2).

**Table 2.** Primary End Points Indicating the Efficacy of Ethosuximide

Measure	ITT population	mITT population	PP population
Patients analyze, No.			
Ethosuximide	64	46	28
Placebo	60	53	41
Responders, No. (%) [95% CI]^a^			
Ethosuximide	17 (26.6) [15.7 to 37.4]	15 (32.6) [19.1 to 46.1]	15 (53.6) [35.1 to 72.0]
Placebo	14 (23.3) [12.6 to 34.0]	10 (18.9) [8.3 to 29.4]	10 (24.4) [11.2 to 37.5]
Relative risk (95% CI)	1.14 (0.61 to 2.11) /	1.73 (0.69 to 4.35)	2.20 (1.15 to 4.18) /
NNT (95% CI)	NA	7.3 (−3.2 to 29.2)	3.4 (1.9 to 15.4)

Abbreviations: ITT, intention-to-treat; mITT, modified intention-to-treat; NNT, number needed to treat.

^a^ Responders was defined as a combination of −30% pain intensity and Subject Global Assessment of Relief score at 4 out of 5 at the end of treatment.

### Secondary Analyses

Secondary outcomes should be interpreted cautiously as exploratory analyses. Multivariate analyses confirmed the findings of no significant difference in responder rates between groups (mITT: RR, 1.62; 95% CI, 0.80-3.27; PP: RR, 2.15; 95% CI, 1.14-4.04; *P* = .02). Apart from treatment group in the PP analysis, no other patient characteristics (sex, age, IBS subtype, or IBS duration) were significantly associated with responder status (eFigure in Supplement 2).

The proportion of patients achieving an SGA score of at least 4 out of 5 did not differ significantly between groups in the ITT population: 16 of 64 patients (25.0%; 95%CI, 14.4%-35.6%) in the ethosuximide group vs 16 of 60 patients (26.7%; 95% CI, 15.5%-37.9%) in the placebo group (RR, 0.94; 95% CI, 0.52-1.07). However, a significant difference was observed in the PP population: 17 of 28 patients (60.7%; 95% CI, 40.7%-77.9%) in the ethosuximide group vs 12 of 41 patients (29.3%; 95%CI, 16.6%-45.7%) in the placebo group (RR, 2.07; 95% CI, 1.82-5.23; *P* = .01). For a reduction of at least 30% in abdominal pain intensity at the end of treatment, no significant difference was observed in the ITT population: 17 of 64 patients (26.6%; 95% CI, 15.7%-37.4%) in the ethosuximide group vs 22 of 60 patients (36.7%; 95% CI, 24.5%-48.9%) in the placebo group (RR, 0.72; 95% CI, 0.43-1.23), with similar findings in the PP population (eTable 1 and eTable 2 in Supplement 2).

Overall, secondary end points (IBS-SSS, GIQLI, and EQ-5D-3L scores) did not differ between groups in either the ITT or PP analyses. Nevertheless, significant within-group improvements from baseline to the end of treatment were observed across both groups (eTable 1 and eTable 2 in Supplement 2).

#### Safety

Patients in the ethosuximide group received lower mean daily doses (7.5 mL/d) and had shorter treatment durations (8.6 weeks) compared with the placebo group (10 mL/d and 9.9 weeks), reflecting reduced tolerability that led to treatment discontinuations or dose reductions. In the ethosuximide group, 30 patients (46.9%) discontinued treatment early because of AEs (15 patients [23.4%]), personal choice (12 patients [18.8%]), or other reasons (3 patients [4.7%]). Discontinuation was significantly lower in the placebo group (13 patients [21.7%]; *P* = .003), including for AEs (6 patients [10.0%]), personal choice (5 patients [8.3%]), or other reasons (1 patient [1.7%]).

AEs occurred in 55 patients (85.9%) in the ethosuximide group (261 AEs total) and in 46 patients (76.7%) in the placebo group (202 AEs total). Among AEs in the ethosuximide group, 158 (60.5%) were mild, 80 (30.8%) were moderate, and 23 (8.7%) were severe; 2 AEs were serious (severe dizziness and allergic reaction to a horsefly bite). Among all 261 AEs in the ethosuximide group, 93 (36.0%) were considered treatment related. In the placebo group, 99 AEs (48.9%) were mild, 91 AEs (45.3%) were moderate, and 12 AEs (5.8%) were severe; 2 were serious (hospitalization for increased pain and allergic skin rash). Among all 202 AEs in the placebo group, 64 (31.7%) were deemed treatment related. The most common treatment-related AEs in the ethosuximide group were headaches or migraines (23 AEs [24.7%]), sleep disturbances (12 AEs [12.9%]), fatigue (11 AEs [11.8%]), nausea (9 AEs [9.7%]), abdominal pain (9 AEs [9.7%]), and dizziness (6 AEs [6.5%]). A similar AE profile was observed in the placebo group, most frequently headaches or migraines (18 AEs [28.1%]), fatigue (10 AEs [15.6%]), nausea (9 AEs [14.1%]), and dizziness (7 AEs [10.9%]) (Table 3).

**Table 3.** Reported AEs by Group

Outcome	No. (%)		*P* value^a^
	Ethosuximide (n = 64)	Placebo (n = 60)	
Treatment discontinuation			
All	30 (46.9)	13 (21.7)	.003
Caused by AEs	15 (23.4)	6 (10.0)	.046
Patient decision	12 (18.8)	5 (8.3)	.09
Others	3 (4.7)	1 (1.7)	.62
Patients reporting AEs	55 (85.9)	46 (76.7)	.31
AEs reported (n = 463)	261 (56.4)	202 (43.6)	<.001
Mild	158 (60.5)	99 (48.9)	.01
Moderate	80 (30.8)	91 (45.3)	.001
Severe	23 (8.7)	12 (5.8)	.25
Treatment-related AEs (n = 261)^b^	93 (36.0)	64 (31.7)	.37
Serious AEs	2/261 (0.8)	2/202 (1.0)	1.0
Treatment-related AEs , No./total No. of AEs reported by >5% of patients (%)			
Headaches or migraines	23/93 (24.7)	18/64 (28.1)	.63
Sleep disorders	12/93 (12.9)	3/64 (4.7)	.15
Tiredness	11/93 (11.8)	10/64 (15.6)	.49
Nausea	9/93 (9.7)	9/64 (14.1)	.40
Abdominal pain	9/93 (9.7)	3/64 (4.7)	.36
Dizziness	6/93 (6.5)	7/64 (10.9)	.32

Abbreviations: AE, adverse event.

^a^ χ^2^ or Fisher tests between ethosuximide and placebo groups.

^b^ According blind expertise by investigator.

Overall, ethosuximide was associated with a higher rate of discontinuations due to AEs than placebo (15 discontinuations [23.4%] vs 6 discontinuations [10.0%]; *P* = .046). The severity of AEs was comparable between groups, suggesting that the higher discontinuation rate in the ethosuximide group was primarily related to AE occurrence rather than intensity.

## Discussion

This randomized clinical trial evaluated the efficacy and safety of ethosuximide, a T-type calcium channel blocker, for the management of IBS-related abdominal pain. Among 124 randomized patients, no statistically significant difference in responder rates was observed between ethosuximide and placebo in the ITT or mITT analyses. In contrast, the PP analysis showed a significantly higher responder rate in the ethosuximide group. However, the absence of significance in the ITT and mITT populations limits the generalizability of this finding. The discrepancy likely reflects the higher discontinuation rate in the ethosuximide group, primarily due to its limited tolerability. Taken together, these results suggest that ethosuximide cannot be recommended for the treatment of IBS-related abdominal pain because of its poor tolerability and modest efficacy.

Our results do not confirm previous preclinical and early clinical data supporting T-type channel inhibition for chronic pain. Experimental studies have consistently demonstrated the central role of these channels, particularly the Cav3.2 isoform, in pain modulation, and inhibition of these channels has been shown to reduce visceral hypersensitivity in animal models.^11,12^ In humans, Cav3.2 overexpression has been observed in the colonic mucosa of patients with IBS,^13^ and a prior clinical study of ethosuximide in this population suggested a potential therapeutic benefit.^14^ However, that study’s open-label, uncontrolled design limited its interpretability. Our randomized clinical trial did not confirm these findings, primarily due to the poor tolerability of ethosuximide. Nonetheless, these results should not discourage further exploration of T-type channels blockade as a therapeutic strategy for chronic pain. Rather, our findings underscore the need to develop more selective and better-tolerated T-type calcium channel modulators.

In clinical practice, recommendations for the first-line management of IBS-related abdominal pain differ among the American College of Gastroenterology,^31^ the American Gastroenterological Association,^32^ and the British Society of Gastroenterology.^33^ Neuromodulators, particularly tricyclic antidepressants, such as amitriptyline, are commonly recommended. The ATLANTIS trial^34^ demonstrated low-dose amitriptyline (10-30 mg per day) significantly improved IBS severity, global assessment, and pain scores with a good tolerability at 6 months. Similarly, a meta-analysis of 4 clinical trials confirmed the therapeutic benefit of amitriptyline.^35^ In comparison, ethosuximide appears to have a less favorable efficacy-tolerability profile. Nevertheless, the greater analgesic response observed in the PP analysis raises the possibility that patient stratification based on tolerability or predictive biomarkers could improve treatment outcomes. Our study did not identify a specific responder phenotype based on sociodemographic or clinical characteristics, suggesting the need to explore additional determinants of response. A promising avenue involves genetic variations in cytochrome P450 (*CYP3A*), the primary enzyme responsible for ethosuximide metabolism.^36^ Comparing *CYP3A* genotypes between responders and nonresponders could yield valuable insights for personalized approaches.

### Limitations

This study has some limitations. The principal limitation of this study was the high dropout rate in the ethosuximide group due to poor tolerability, resulting in less than half of participants completing treatment in the PP population. This likely would lead to overestimated efficacy, as the PP analysis reflects only patients who could tolerate the treatment. Therefore, these results should be interpreted as exploratory. Additionally, we did not assess visceral sensitivity using a distension test, quantify Cav3.2 expression in the colonic mucosa, or measure plasma ethosuximide concentrations. Such data could have provided mechanistic insights, facilitated baseline phenotyping (eg, identifying visceral hypersensitivity or Cav3.2 overexpression), and helped characterize responders and nonresponders. However, these assessments require specialized equipment and expertise not uniformly available across participating centers.

## Conclusions

This randomized clinical trial found no significant benefit of ethosuximide for the treatment of IBS-related abdominal pain. Future research should focus on developing more selective Cav3 antagonists with improved safety and identifying predictive biomarkers to optimize patient selection. Integrating pharmacogenetics and objective measures of visceral hypersensitivity may enhance therapeutic precision and guide individualized treatment strategies in IBS.

## Notes

Supplement 1.
Trial Protocol and Statistical Analysis Plan

Supplement 2. **eFigure.** Forest plot of relative risk comparing responder status with patient characteristics and study treatments **eTable 1.** Secondary efficacy end points: ITT population **eTable 2.** Secondary efficacy end points: PP population

Supplement 3.
The IBSET Investigator Group (IIG)

Supplement 4.
Data Sharing Statement
