# Supplementary material for: Ethosuximide and Irritable Bowel Syndrome–Related Abdominal Pain: A Randomized Clinical Trial
Source: JAMA Netw Open. 2026 Jan 8;9(1):e2551368. doi: 10.1001/jamanetworkopen.2025.51368 (PMC12784227; doi:10.1001/jamanetworkopen.2025.51368)
Supplement: Supplement 1. — Trial Protocol and Statistical Analysis Plan [file jamanetwopen-e2551368-s001.pdf]

**RESEARCH PROTOCOL (SPIRIT GUIDELINES)**

**Administrative Information**

Title: *Assessment of the Effectiveness and Safety of Ethosuximide in the Treatment of Abdominal Pain related to Irritable Bowel Syndrome – IBSET: protocol of a randomised, parallel, controlled, double-blind and multicentre trial.*

Acronym: IBSET

Trial registration:
Sponsor code: PHRC IR 2015 DAPOIGNY
EudraCT number: 2016-002110-42
Clinical trial registration: NCT 02973542

Protocol version:
Version 4 (28/11/2016)

Funding: This work is supported by funding from the French Ministry for Health ("Programme Hospitalier de Recherche Clinique Interrégional", year 2015) and the SFETD ("Société Française d'Etude et de Traitement de la Douleur").

Roles and responsibilities:

**SPONSOR:** C.H.U. de Clermont-Ferrand, 58 Rue de Montalembert, 63003 Clermont-Ferrand, FRANCE
Patrick Lacarin, placarin@chu-clermontferrand.fr
Roles and responsibilities: management, analysis and writing of the report

**COORDINATING INVESTIGATOR AND CENTRE :**

**Pr. Michel DAPOIGNY :** mdapoigny@chu-clermontferrand.fr +33 (0)4 73 75 05 28
UMR INSERM / UdA 1107 NEURO-DOL, University of Auvergne, 63000 Clermont-Ferrand, France.
Gastroenterology service, University Hospital Estaing, 63000 Clermont-Ferrand, France.
Roles and responsibilities: Study design, interpretation of data, data management team, monitoring

**INVESTIGATORS AND TRIAL CENTRE :**

| | |
|---|---|
| **Pr. Claude DUBRAY**<br>CHU Gabriel-Montpied, 63000 Clermont-Ferrand, FRANCE | **Pr. Jean-Marc SABATE**<br>CHU Louis Mourier-AP HP, 92701 Colombes, FRANCE |
| **Pr. Thierry PICHE**<br>CHU Nice, 06202 Nice, FRANCE | **Pr. Bruno BONAZ**<br>CHU Grenoble, 38000 Grenoble, FRANCE |
| **Dr. Camille SAUTEL**<br>CH Issoire, 63500 Issoire, FRANCE | **Pr. Véronique VITTON**<br>CHU Marseille, AP-HM, 13915 Marseille, FRANCE |
| **Dr. Sabine ROMAN**<br>CHU Lyon, HCL, 69437 Lyon | **Dr Julien SCANZI**<br>CH Thiers, 63300 Thiers |
| **Pr. Philippe DUCROTTE**<br>CHU Rouen, 76031 Rouen | |

| Methodologist / Statistics : | Scientific collaborators : |
|---|---|
| **Dr. Bruno PEREIRA**<br>DRCI, CHU Clermont-Ferrand 63000 Clermont-Ferrand, FRANCE | **Pr. Denis ARDID / Dr. Nicolas KERCKHOVE**<br>UMR Inserm / UdA 1107 NEURO-DOL<br>CHU Gabriel-Montpied, 63000 Clermont-Ferrand, FRANCE |

**Introduction**

Irritable bowel syndrome (IBS) is characterized by abdominal pain, bloating and changes in bowel habit, in the absence of an identifiable organic disease [1,2]. This functional disorder is very common as its prevalence is estimated around 10%-15% in Europe and the United States [3–6]. IBS chronicity severely impacts patients' quality of life and generates large healthcare costs [7,8]. The high prevalence and the absence of effective treatments make this disease a public health concern. The mechanisms involved in visceral pain and IBS symptoms are still unknown. Recent studies indicate that alterations of the intestinal epithelial barrier is a key pathophysiological event in IBS, allowing maintenance of a low grade mucosal inflammatory state, leading to visceral hypersensitivity (VH) and pain [9,10]. This VH, highlighted by the colorectal distension test [11], is found in more than half of IBS patients [12–14]. Hypersensitivity implies sensitization of somatic or visceral primary afferent fibres, involving an increase in excitability. In several animal models of chronic pain, this hyperexcitability has been linked to a remodelling of ion channel expression patterns [15,16]. Several voltage-gated ion channels are involved in neuronal firing, including T-type calcium channels, especially Cav3.2 member [17,18]. We recently found in a case-control study that this Cav3.2 channel was overexpressed in the colonic mucosa of IBS patients compared to asymptomatic controls [19]. Moreover, it has been shown that the blockade of Cav3.2 channels, in an animal model mimicking IBS, prevented the development of colonic hypersensitivity [20]. We aimed to evaluate the efficacy and the safety of a T-type calcium channel blocker –ethosuximide- in the treatment of abdominal pain in IBS patients.

**T-type calcium channels, ethosuximide and IBS:**
For ten years, Cav3.2 channels have been extensively studied and shown as involved in nociceptive pain [18,21]. Marger *et al.* recently demonstrated, in an animal model of butyrate-induced VH , that Cav3.2 channels were involved in chronic visceral pain [20]. They have shown that Cav3.2 channels were overexpressed in the dorsal root ganglion neurons innervating the colon and that their pharmacological blockade prevented the development of colonic hypersensitivity. Recently, we demonstrated in a case-control clinical study, that Cav3.2 channels were overexpressed in the colonic mucosa of IBS patients compared to asymptomatic controls [19]. These channels are located particularly in peripheral nerve fibres, supporting their involvement in the development of colonic hypersensitivity. Cav3.2 channels appear to be an interesting pharmacological target to treat visceral pain related to IBS. The therapeutic effect of ethosuximide was studied in several animal models of chronic pain: traumatic neuropathy [22–24] and chemotherapy-induced peripheral neuropathy [25,26], inflammatory [27–30] and acute pain [29–32]. Moreover, ethosuximide was shown to be a modulator of pro-nociceptive effects of hydrogen sulfide (H2S) [28,33,34], a gazotransmitter involved in VH related to IBS [35,36] and a Cav3.2 channel activator [37,38]. Thus, according to the results of these studies, ethosuximide, by its action on T-type calcium channels, seems to be a promising and innovative therapeutic in the treatment of abdominal pain in IBS patients.

<u>Summary of hypotheses and objectives:</u>
T-type calcium channels blockers have a major interest for the development of new symptomatic treatment of pain related to IBS. There is an opportunity to conduct an innovative proof-of-concept trial in IBS patients with such a blocker, ethosuximide, which is currently available on the European market (to

treat epilepsy). The demonstration of clinical efficacy would enrich our pharmacopoeia to treat pain related to IBS.

The primary objective of this study is to evaluate the efficacy of ethosuximide versus placebo, administered in addition to background therapy, on intensity of abdominal pain related to IBS and to evaluate the score of the Subject Global Assessment (SGA scale) of Relief.

The secondary objectives concern the effects of ethosuximide on:
- the severity of IBS symptoms,
- the quality of life (physical and mental),
- the safety of ethosuximide.

The study discontinuation rate and analgesics and transit regulator consumption will be assessed throughout the study.

Trial design:
Multicentre, randomized, double-blind, parallel group and controlled pilot clinical trial.
Safety and efficacy.

**Methods**

Study setting: Academic Hospitals

Eligibility criteria:
*Inclusion criteria*

- Man or woman aged 18 or more.
- Negative pregnancy test and effective contraception.
- IBS defined by ROME IV criteria.
- Treatment failure (11-points NRS abdominal pain ≥ 4) for at least 3 months despite stable treatment for a month.
- Patients affiliated to the regime of the French Social Security.
- Patients able to deliver a free and informed consent.

*Exclusion criteria*

- Breastfeeding.
- Diabetic patients.
- Chronic pain of greater intensity than the pain related to IBS.
- Renal or hepatic impairment defined by significant liver function (liver enzymes > 3 x normal values, cholestasis) and renal (MDRD < 60 ml/min) abnormalities.
- History of severe or current depression (hospitalization, long-term antidepressant treatment).
- Addiction to alcohol and/or drugs.
- Patient already treated with antiepileptic.
- Allergy to succinimide (ethosuximide, methsuximide, phensuximide).
- Psychotic disorders.
- Insufficient cooperation and understanding to adhere strictly to the conditions provided by the study.
- Patients undergoing a measure of legal protection.

Interventions:

Study methodology was selected on the recommendation of the European Medicines Agency (ICH guideline Irritable Bowel Syndrome: EMA/CHMP/60337/2013-CPMP/EWP/785/97 Rev. 1; for short treatment on all types of IBS).

Patients will be treated for 12 weeks either by ethosuximide or a placebo, according to a regimen with a specific titration.

*Enrolment*

Patients followed in their referral centre for the treatment of IBS will be pre-selected. Patients will be contacted in order to briefly present the purpose of the study and make an appointment for the inclusion visit.

*Visit 1 - Inclusion (D0-7) and run-in period (D0-7 to D0)*

The objectives of the study, practice organisation, and constraints will be explained in details. The investigator will present the information and consent form. The patient must present an IBS diagnostic defined according to the ROME IV criteria. Two blood samples will be taken (blood count, hepatic enzymes, renal function, β-HCG and genetic analysis).

A daily logbook will be given to the patient with detailed explanations to collect daily, every week before planned visits, the median abdominal pain score experienced during the last 24 hours and the Bristol score. Side effects should be collected. The patient will return home with a logbook for 7 days, corresponding to the run-in period. This run-in period, without any studied treatment, was introduced (i) to evaluate the ability of the patients to rate their abdominal pain daily in their logbook; (ii) to check the basal average pain intensity over a 1-week period. The patients received the treatment after this run-in period only if the inclusion criteria were met (11-points NRS abdominal pain ≥ 4 and completed logbook as required by the protocol).

*Visit 2 - Start treatment (D0)*

Daily average abdominal pain scores have to be filled-in by the patient on the logbook during the last 7 days and the average abdominal pain score should be ≥4 to include the patient in the study. The patients have to fill-in three questionnaires: 1) IBS-SSS, 2) EQ-5D and 3) GIQLI.

If all the inclusion and non-inclusion criteria are conformed, the patient will be enrolled in the study and randomised in one of the two treatment arms (ethosuximide or placebo). The administration dosages, according to a specific titration regimen, will be explained in details to the patient. At the end of the visit, the patient will receive all the therapeutic units for the duration of administration required by the study protocol (4 weeks) and the daily logbook.

*Treatment period 1 - Dose escalation - (D0 to D0+4 weeks)*

Ethosuximide or placebo treatment will be administered daily during dinner, according to a regimen of specific titration for 28 days (maximum dose of 10 ml/day). The patient will assess and record the following daily in the logbook:

- The evening before bed: abdominal pain felt in the last 24 hours with the 11-points NRS,
- The evening before bed: the number, shape and consistency of the stool with the Bristol's scale,
- Side effects or any other particular event should also be carefully noted in the daily logbook,
- Treatment modification.

Every 7 days (at the end of each dose escalation level), patients will be contacted by phone call by investigator in order to collect information on any side effects and inform the patient of the increased dose

in case of inefficiency and good tolerance. Stopping or decrease dosage will be used in cases of intolerance or effectiveness of the treatment.

*Visit 3 – follow-up visit (D0+4 weeks)*

This first visit will provide information on the therapeutic dose with an optimal benefit-risk ratio for the patient.

During the visit, the patient will complete questionnaires IBS-SSS, EQ-5D, GIQLI questionnaires and the SGA scale. At the end of the visit, the patient will receive all therapeutic units for the next treatment period prescribed by the protocol (4 weeks) and the logbook.

*Treatment period 2 and 3 (D0+4 weeks to D0+8 weeks and D0+8 weeks to D0+12 weeks)*

Likewise treatment period 1. To note that the patient will assess and record, only the 7 days before the next visit, the following in the logbook:

- The evening before bed: abdominal pain felt in the last 24 hours with the 11-points NRS,
- The evening before bed: the number, shape and consistency of the stool with the Bristol's scale.

*Visit 4 and 5 – follow-up visit (D0+8 weeks and D0+12 weeks)*

Likewise visit 3. Visit 5 closing the treatment period.

To note that at the end of visit 5, will be given to the patient the abdominal pain 11-points NRS to complete daily for 7 days, 3 weeks after the visit 5 (± 1 week).

*Phone call follow-up to D0+15 weeks (± 1 week)*

This phone call aims to recover daily scores of abdominal pain during the last 7 days. This phone call closes the patient's study participation.

- All patients followed the same procedures (assessment of pain, filling questionnaires and taking treatment in the indicated dosage level).
- No deviation to the protocol will be allowed during the entire study.
- To verify patient compliance, accounting treatment units will be made at each planned visits.
- No change of treatment will be allowed during the test.

<u>Outcomes:</u>

Study endpoints were selected on the recommendation of the European Medicinal Agency (ICH guideline IBS: EMA/CHMP/60337/2013-CPMP/EWP/785/97 Rev. 1: for short treatment on all types of IBS).

*Primary endpoint*

Rate of responders to treatment; a patient will be considered as a responder if he satisfies two conditions: 1) a decrease of at least 30% of the score of abdominal pain (11-points NRS) compared to the score before treatment, and 2) a significant global improvement defined by a score of 4 or 5 on the SGA scale.

<u>Abdominal pain intensity (11-points NRS):</u> This scale allows the patient to rate his abdominal pain from 0 to 10; with 0 is no pain and 10 the worst possible abdominal pain. The intensity of abdominal pain (the past 24 hours) will be measured daily (the evening) by the patient on the logbook, and the values will averaged for the 7 days preceding the two time points D0 (baseline) and D0+12 weeks (last visit). The primary endpoint is the calculated difference $\Delta(\%) = ((NRS(D0) – NRS(D0+12\ weeks)/\ NRS(D0)) \times 100$.

Subject Global Assessment of Relief: The Subject Global Assessment of Relief [39] aims to assess the effectiveness of a specific treatment on symptoms of IBS. This scale consists of 5 levels of description answering the question "Please consider how you felt during the past treatment period in regard to your IBS, in particular your overall well-being, and symptoms of abdominal pain/discomfort and altered bowel

habit". Levels of description are declined such as: 1) worse, 2) not at all relieved, 3) somewhat relieved, 4) considerably relieved and 5) completely relieved".
These simple responses sign both satisfaction and dissatisfaction. This evaluation will be conducted during the last visit of the study (D0+12 weeks).

*Secondary endpoints*
Health Related Quality of Life (GIQLI and EQ-5D-3L): HRQoL will be evaluated by the Gastrointestinal Quality of Life Index (GIQLI) and EuroQol five dimensions questionnaires (EQ-5D-3L, EuroQol group) which assess the physical and mental health of IBS patient.
The GIQLI questionnaire [40] is a specific self-questionnaire for measuring the quality of life of patients with gastrointestinal disease. The questionnaire includes 36 items asking about symptoms, physical status, emotions, social dysfunction, and effects of medical treatment.
The quality of life questionnaire "EQ-5D" [41] is a non-specific questionnaire used to assess the health status and the impact of a disease on patient's quality of life. The EQ-5D score is measured on 5 dimensions (mobility, personal autonomy, routine activities, pain and anxiety/depression) with three levels per dimension and a scale from 0 (worst imaginable state of health) to 100 (best imaginable state of health).
These assessments are done at every planned visit.

IBS severity (IBS-SSS and Bristol scale): IBS-SSS questionnaire [42] is a self-administered questionnaire used to assess the severity of the specific symptoms related to IBS. This questionnaire consists of 7 questions giving a score between 0 and 500 points. IBS intensity is considered mild for a Francis score less than 175, moderate for a score from 175 to 300 and severe if the score is above 300.
The scale of Bristol is a medical diagnostic tool created to classify the shape and consistency of human faeces. The scale has seven distinct categories. This assessment is done at home by the patient alone, every week before planned visits, on the daily logbook (the evening (before bed): stools number and consistency).

Genetic analysis of cytochromes P450:
A blood sampling (1 tube of 6 ml) will be performed for the genetic analysis of the genes coding for CYP3A4 and CYP2E1, involved in the metabolism of ethosuximide. This genetic analysis will make it possible to demonstrate the existence (or not) of slow / fast metabolizers in our studied population. The demonstration of slow / rapid metabolizers will enable us to carry out a more precise analysis on the therapeutic efficacy of ethosuximide.
These blood samples will be strictly used in this study and a specific document informing the patient and obtaining his authorization will be provided during the first visit (D0-7).

Analgesic and antispasmodic/regulators transit consumption: Drugs consumption for IBS will be evaluated throughout the study

Safety: Any adverse events were collected daily by the patient and during the planned phone calls by investigators (every 7 days). Adverse events are categorized according to their type, intensity and treatment related by investigators. Due to the long half-life of ethosuximide (60h), a phone call, within 3 weeks after stopping treatment, will be allowed to recover any adverse effects.
Study discontinuation rate will be evaluated and compared in the two treatment arms.

Participant timeline (see figure 1):
The duration of treatment is 12 weeks. The total duration of patient participation is maximum 16 weeks. The protocol includes 5 visits (Day0-7, Day0, Day0+4 weeks, Day0+8 weeks and Day0+12 weeks).

Sample size:
According to previous work presented in literature[43], we have estimated that a sample size of n = 130 patients per randomized group, for a two-sided type I error at 5%, would provide 90% statistical power of detecting an absolute difference of 20% between ethosuximide and placebo in the primary outcome: rate of responders to treatment as defined previously (35% for placebo and 55% for ethosuximide). Finally, a total of n=290 patients (145 by group) will be considered to take into account lost to follow-up (10%). An interim analysis will be performed after 100 included patients to analyse safety and effectiveness in order to recalculate the sample size according results.

Enrolment:
At least ten clinical sites participate in France, all specialized in the treatment of IBS. The recruitment will be spread over 30 months with 1 patients/month/centre, which seems to be largely sufficient in the recruitment potential (prevalence of IBS = 15% in French population [[4]]).

Assignment of interventions:
Allocation Sequence generation: computer-generated random numbers. Stratification by centre and 6 blocks random sequence.
Allocation concealment: sequentially numbered.
Implementation: Biostatistician generates the allocation sequence, investigators enrol participants and Clinical Research Associate (CRA) assigns participants to interventions.

Blinding:
All participants to study are blinded (double-blind trial). Unblinding is possible only in case of serious adverse event or at the end of study if the study treatment showed a convincing therapeutic effect (Possibility of prescribing the treatment out of the study).

Data collection, management, and analysis:

*Data collection methods*
Data entry will be made by the investigators and CRA of each clinical centre, at each planned visits and during the ambulatory period.
Data entry will be centralized in the coordinating centre (CHU Clermont-Ferrand), from an electronic case report form (e-CRF, Ennov Clinical, France) filled-in during site visits.
All patients will be analysed (intent to treat analysis). However, if a major deviation from the protocol (non-respect of co-occurring treatment, inclusion / exclusion criteria and the study design) only safety data will be evaluated.

*Data management*
A consistency test will be conducted to validate compliance of data entered in accordance with the study protocol.

*Statistical methods*

Statistical analyses will be conducted using Stata software (version 13, StataCorp, College Station, US). A two-sided p-value of less than 0.05 will be considered to indicate statistical significance (except interim analysis).
Intent-to-treat (ITT), mITT, and per-protocol (PP) analyses were performed for the primary endpoint and expressed as relative risks (RR) with 95% confidence intervals (CI). Non-compliant patients were considered non-responders. Responder rates were compared using the chi-squared test and a pre-specified multivariate analysis was performed using a generalized linear mixed model (Poisson regression with a log link and robust variance 26) accounted for fixed covariates (e.g., gender, IBS subtype) and centre-level variability were performed. Primary endpoint components were also analysed with the Hochberg procedure for multiple testing27.
Continuous outcomes were assessed with linear mixed models incorporating patient-level random effects (slope and intercept) nested within centre-level random effects. Results were expressed as regression coefficients (standardized mean differences - MD) with 95% CI, based on the interaction between time and randomization group. The normality of residuals from these models was analysed as aforementioned. Safety analysis was conducted with chi-squared or Fisher's exact tests.
Missing data (if any) were imputed using Multiple Imputation by Chained Equations (MICE). Analyses were conducted using Stata 15.0, with a two-sided P value <0.05 considered significant, and the widths of confidence intervals were not adjusted for multiplicity and should not be used as a substitute for hypothesis testing, except for the primary outcome and its composite components. An interim analysis is planned after enrolment of the first 100 patients using the Lan and DeMets rule (East software, Cytel Inc, Cambridge, Massachusetts, US). The type I error is fixed at 0.03 for this interim analysis. In addition, an early intermediate analysis (third of the inclusions) will be also proposed in order to study safety of the initial maximum dose (10 ml/day) and, if necessary, to allow the addition of a therapeutic dose in case of inefficiency.
According to clinical relevance and to EMA and CONSORT recommendations, sub-group analyses depending on IBS subgroup and gender will be proposed after the study of sub-group x randomization group interaction in regression models (for repeated data or not). A descriptive analysis of stopping and discontinuations in view of dose escalation will be considered. A particular focus will be done to safety and lose to follow-up. A study of abandonment considered as censored data will be proposed using the Kaplan-Meier estimation.

Monitoring:
The monitoring will be performed by the University Hospital of Clermont-Ferrand which is responsible for establishing the schedule and procedures to be followed for monitoring this study. On-site visits will be made prior to study initiation and at regular intervals during the study. Communications by telephone, telefax or mail may be used as needed to supplement site visits.

Prior to the beginning of this study, the Investigator will be informed as to the anticipated frequency of the monitoring visits. In addition, the Investigator will receive reasonable notification prior to each monitoring visit during the course of the study.
The purpose of these visits is to verify:
- Adherence to the protocol,
- Availability of completed ICFs and adequate consent process
- Completeness and accuracy of the e-CRFs, and study related source document.
At each visit, the Investigator will be expected to cooperate with the monitor for the review and verification of all e-CRFs, the study drug supply and inventory records and any additional records as may have been previously arranged.

At the pre-study visit, the study monitor and/or the Sponsor representative will check that the Investigator has the technical means and the staff to carry out the study with regards to availability, subject recruitment, facilities and environment.

Prior to the start of the study, the Sponsor Study Manager will ensure that he/she has received the following information:
- Study protocol and financial agreement signed by all parts;
- Written statement of the CPP approval;
- Curriculum vitae of the investigators;
- Approval by the Competent Authority; ANSM.
As well as all other documents required for study initiation, as per GCP.

During the study, adherence to the protocol, availability of signed ICFs and conformity of the data entered in the e-CRF with the source documents will be checked at appropriate intervals by the study monitor.

At the end of the study, the Sponsor study manager will ensure he has received:
- The completed e-CRFs;
- All unused medications and remaining packaging, or those they have been destroyed in accordance with the applicable regulations.
- All documents are properly filed in the Investigator's site file as per GCP.

**Ethics and dissemination**

Research ethics approval:
The protocol, information and consent form (ICF) and the e-CRF of the study will be submitted for opinion to the ethic committee (CPP VI of the Rhône-Alpes-Auvergne region) which carries the principal investigator of this trial. Notification of the approval of the CPP will be sent to the study sponsor and competent authority (ANSM). An authorization request will be made by the promoter to ANSM before the start of the study.
When necessary, the protocol amendments will also have to be submitted to the above mentioned CPP or ANSM either for information or for formal approval. With the exception of emergency situations, no changes or deviations in the conduct of this protocol will be permitted without the documented approval of the Sponsor. The IEC as well as the French Health Authorities which granted original approval for the study must be notified of all changes in the protocol and must provide documented approval for any change or deviation which may increase the risk to the subject and/or which may adversely affect the rights of the subject or validity of the investigation. This stipulation does not apply to those changes made to reduce discomfort or risk to subjects or which are purely administrative in nature.
In the event of any emergency, the Investigator shall institute any medical procedures, which he/she deems appropriate. However, all such procedures must be promptly reported to the Sponsor.
Amendments that are substantial and are likely to have an impact on the safety of trial subjects, or are otherwise significant, should receive IEC/Competent Authority approval. If the opinion of the IEC is favourable and the Competent Authorities have raised no grounds for unacceptability the study can be conducted according to the amended protocol. Non substantial amendment should only be notified.

Consent or assent:
Voluntary written ICF must be obtained from each subject prior to performing any study related procedures in compliance with the recommendations of the Declaration of Helsinki.

Subject should not be screened or treated until the subject has signed an approved ICF written in a language that is understandable to the subject.
Each subject should be given both verbal and written information describing the nature and duration of the clinical study. The ICF should be signed and personally dated in two originals by the subject and the person who conducted the informed consent discussion. The
Investigator, or the attending physician, will explain the nature, purpose and risks of the study. The subject will be informed that he has the right to withdraw at any time from the study, without giving reasons. In this case, the subject will not receive any indemnity. The informed consent process should take place under conditions where the subject has adequate time to consider the risks and benefits associated with his participation in the study.
The Investigator is responsible for assuring the appropriate content of the ICF and that informed consent is obtained from each subject in accordance with all applicable regulations and guidelines. The ICF will be reviewed and approved by the Sponsor, and then submitted to the IEC.
Each subject should receive one original of the signed and dated written ICF and any other information provided to the subject.
The second original of the signed and dated ICF should be retained in the Investigator's file.
The Investigator should maintain a log of all subjects who sign the ICF.

Confidentiality:
The information in this document and in any future information supplied contains trade secrets and commercial information that are privileged or confidential and may not be disclosed unless such disclosure is required by law or regulations.
In any event, persons to whom the information is disclosed must be informed that the information is privileged or confidential and may not be further disclosed by them.
The investigator must assure that subjects' anonymity will be maintained and that their identities are protected from unauthorized parties. On e-CRFs or other documents submitted to the sponsor, subjects should not be identified by their names, but exclusively by an identification code.

Declaration of interests:
No competing interests.

Access to data:
The promoter is responsible for obtaining the agreement of all parties involved in research in order to guarantee direct access to all places of conduct research, source data, source documents and reports in a goal quality control and audit by the sponsor.
The investigators will make available documents and individual data strictly necessary monitoring, quality control and auditing of biomedical research, available to people with access to these documents in accordance with legislative and regulatory provisions (Articles R.5121-13 L.1121-3 and the code of public health).

Ancillary and post-trial care:
If the patient is at the waning of the study indicates a marked improvement in his symptoms, unblinding procedure will be permitted to determine the drug to be prescribed after the study. Unblinding procedure, randomization plan and packaging of therapeutic units will maintain intact the double blind until the end of the study. Patients will be referred to their general practitioner or a specialist pain doctor who will be informed of the treatment received by the patient during the study. Note that the requirement in Zarontin® or placebo for the treatment of pain related to IBS being out of their marketing authorization framework,

the sponsor does not support a continuation of treatment after the study. No patient follow-up is planned after the study.

Dissemination policy:

The data set will be propriety of the sponsor (CHU Clermont-Ferrand). However, the principal investigator (MD) and the project manager (NK) will have a full access to the final data set. The results will be published in a peer-reviewed journal, presented at international congresses and completed online on ClinicalTrials.gov.

APPENDICE 1 – INFORM AND CONSENT FORM

**Assessment of the Effectiveness and Safety of Ethosuximide in the Treatment of Abdominal Pain related to Irritable Bowel Syndrome**


**<u>Sponsor</u>**

University Hospital of Clermont-Ferrand
58 Rue de Montalembert,
63000 Clermont-Ferrand, France

**<u>Principal investigator</u>**

Pr Michel DAPOIGNY
Gastroenterology service
University Hospital Estaing
63000 Clermont-Ferrand, France


Dr ......................................... has offered to participate in a clinical research protocol, including the University Hospital of Clermont-Ferrand as promoter. All consultations that you will perform as part of this study will be in the gastroenterology department which follows you.
The objective of this study is to evaluate the efficacy of ethosuximide (syrup marketed in France under the name Zarontin®) on the type of abdominal pain that you present.
Like several other drugs widely used to treat abdominal pain, ethosuximide is now prescribed in the treatment of epilepsy in both children and adults.
The study is done in double blind, i.e. you will receive either ethosuximide or a placebo that does not contain an active product. The doctor and you will not be allowed to know about the treatment received.
Finally, you are free to accept or refuse to participate in this search for any reason. In addition, you may exercise your right to withdraw from this research at any time without any justification on your part.

**<u>Enrolment:</u>**

This protocol is proposed to you as well as to 289 other adult patients suffering from chronic abdominal pain. The treatments you usually receive will be continued. No additional treatment for your abdominal pain will be allowed during the entire study (5 visits in 3 months). Nevertheless, in case of excessive abdominal pain, you will be allowed to take analgesics (paracetamol) or antispasmodics (eg phloroglucinol, trimebutine).

**<u>Treatment:</u>**

Treatment (ethosuximide or placebo) will be administered as follows (dose escalation in the first weeks):
- 2.5 ml / day in the evening during the meal for 7 days,
- 5 ml / day in the evening during the meal for 7 days,
- 7.5 ml / day in the evening during the meal for 7 days,
- 10 ml / day in the evening during the meal for 63 days.

Doses of syrup should be taken using a graduated plastic pipette that will be delivered to you. You will not be allowed to stop treatment without prior notice from the investigating physician. In case of adverse events, you will have to contact as soon as possible the investigating physician who follows you.
It is important to know that this dose escalation is theoretical. In case of adverse event or a beneficial effect of the treatment on your symptoms, the dosage may be stop or decreased at the appropriate dose level (with good tolerance and / or with beneficial effect). This decision will be made with the investigating physician during the planned phone calls provided for this purpose or at any time during the study.

**Evaluations:**

Numeric rating scale:
Using a numeric rating scale from 0 to 10, each day during the week preceding your visits, before bedtime, you should note on your logbook the intensity of the average abdominal pain felt during your day (last 24 hours). A score of 0 corresponding to no pain and 10 to an unbearable pain.

Bristol Scale:
Using an illustrated scale, each day during the week before your visits, before bed, you should note on your logbook the number and consistency of your stools.

Questionnaires on the characteristics of your abdominal pain and its impact on your daily life:
The questionnaires will be presented and explained by the investigating physician so that you can fill them out with understanding. These questionnaires are: GIQLI and EQ-5D to assess your quality of life, SGA to assess your feelings about the effectiveness of treatment and Francis to assess the intensity of your irritable bowel syndrome.
They should be completed on each of your 5 visits.

Information about your treatments:
During each visit and during the filling of your logbook, you will be asked to write and inform the investigating physician about all your treatments taken during the study.

Biological assays:
A blood sampling (2-3 tubes, ≈ 15 ml) will be performed during your first visit to the gastroenterology department to perform your blood tests (haematocrit, liver / kidney function and β-HCG for women of childbearing age). These samples will be taken by an authorized nurse.
A second blood sample (1 tube of 6 ml) will be performed for a genetic analysis of the genes CYP3A4 and CYP2E1 (genes involved in the metabolism of study treatment - ethosuximide). This genetic analysis will assess how ethosuximide is metabolized (processed, eliminated) in your body. Possible genetic variations between participants may explain the differences in therapeutic efficacy (if demonstrated) observed.

**Study design:**

As part of this study, you will be required to participate in 5 visits to the gastroenterology department plus a follow-up phone call at 3 weeks (± 1 week) after discontinuing treatment for a 16-weeks study period. During these different visits you will have to complete various questionnaires. Between each visit, you will have to take daily treatment (ethosuximide or placebo) during 12 weeks of treatment (3 periods of 4 weeks).

During these treatment periods, you will also be asked to fill in your logbook daily and each week before your visits to the investigating centre. On this logbook you should indicate the intensity of abdominal pain as well as the number and consistency of your stool. You will have not to change any of your usual treatments throughout the study. However, if it is necessary, it is imperative to inform the investigating physician who follows you during this trial, any changes in the treatments you take regularly or the introduction of new drugs taken or modified.

Recruitment:

You will be selected and contacted by phone by the investigating physician of the gastroenterology department in order to present briefly the purpose of the study and to make an appointment for the first inclusion visit.

Study Summary:

The study you are participating in will take place as described in the table below.

| Visits | Pre-inclusion visit (V1) | Run-in periode | Inclusion visit (V2) | Treatment Periode 1 | Follow-up visit (V3) | Treatment Periode 2 | Follow-up visit (V3) | Treatment Periode 3 | End of treatment (V5) | Follow-up phone call (Study end) |
|---|---|---|---|---|---|---|---|---|---|---|
| Day - Place | D0-7 Centre | D0-7 to D0 Home | D0 Centre | D0 to D0+4 wks Home | D0+4 wks Centre | D0+4 to D0+8 wks Home | D0+8 wks Centre | D0+8 to D0+12 wks Home | D0+12 wks Centre | D0+15 wks (± 1 wk) Home |
| Presentation of the information and consent form | + | | | | | | | | | |
| Clinic exam | + | | | | | | | | | |
| Checking your eligibility criteria under consideration | + | | + | | | | | | | |
| Blood sample | + | | | | | | | | | |
| Filling in the different questionnaires (Francis, GIQLI, EQ-5D) | | | + | | + | | + | | + | |
| SGA scale | | | | | + | | + | | + | |
| Treatment (Ethosuximide or placebo) | | | | + | | + | | + | | |
| Filling in the logbook (Pain and stools consistance) | | + | + | + | + | + | + | + | | + |
| Phone calls | | | | + (Every 7 days) | | | | | | + |

**Profits:**

Given the limited duration of treatment prescribed for you in this study, there is no expectation of long-term individual benefit resulting from participation in the study. However, if there is a significant improvement in episodes of abdominal pain during the study participation period, you will be allowed to continue treatment after the study. Since this treatment is already marketed, your doctor or gastroenterologist will be authorized to prescribe it.

**Expected Risks:**

Ethosuximide has been prescribed for the treatment of epilepsy since the 1980s. The dosage regimen chosen for this therapeutic trial is that normally used in children in this indication. In this trial, dose increments were set at 7 days.

Like most drug therapies, it can cause adverse effects, such as:
Frequent: ataxia, dizziness, headache, drowsiness, rash erythematous, urticaria, epigastric pain ** decreased appetite**, abdominal pain **, nausea** and vomiting **.
** These adverse effects are observed only at the beginning of treatment and usually subside quickly or after dose reduction and do not reappear later.

Infrequent: agranulocytosis, aplastic anaemia, eosinophilia, leukopenia, pancytopenia, depression, suicidal thoughts, lethargy, Stevens-Johnson syndrome, DRESS syndrome (Drug Rash Eosinophilia and Systemic Symptoms), anticonvulsant hypersensitivity syndrome, fatigue, irritability, loss of weight.

These make the intake of this drug incompatible with driving without prior notice from your doctor.

In order to avoid the occurrence of these adverse events, it is strongly recommended that you observe the gradual increase of the doses in stages during the first four weeks up to the maximum dose. Phone contacts by the physician / clinical research assistant / nurse every 7 days (at the end of each step) will be used to remind you of dosage changes and any adverse events you may experience. It is also during these phone calls that you can stop increasing the dose of the treatment or decrease it according to your feelings (beneficial effect / adverse events).
Furthermore, in view of the fact that the drug is already on the market, its safety profile is now very well known.

Ethosuximide and placebo have a high concentration of sugar (about 3g for 5ml). For these reasons, this medication is not recommended for people with diabetes or sucrase / isomaltase deficiency.

The rules of asepsis will be rigorously applied during your blood sampling. The volume of blood taken is minimal (≈ 15 ml).

**Treatments authorized and not authorized during the study:**

Treatments authorized during the test:
All treatments of your irritable bowel syndrome correcting a transit disorder and / or anxiolytic treatments. Your treatments should be stable in terms of intake and dose for at least 1 month before you enter the study. Spasfon® and / or paracetamol (Doliprane®, Efferalgan®), which you will need to mention in the follow-up logbook and during phone calls, will be allowed to take a temporary emergency treatment.

Treatments prohibited during the test:
All antalgic treatments, antispasmodic, transit regulator (excluding Spasfon® and paracetamol).

**Exclusion period for participation in other biomedical research:**

You are not allowed to participate in another clinical trial throughout the duration of the protocol.
In addition, the exclusion period defined in this study is 30 days, during which time you will not be able to participate in another clinical research protocol.

The CHU of Clermont-Ferrand, which organizes this biomedical research as a promoter, has contracted insurance in accordance with the legal provisions, guaranteeing its civil liability and that of any intervener with the Société Hospitalière d'Assurances Mutuelles (SHAM, contract no. 147161).
In case your health condition is altered as a result of your participation in the study, in accordance with Public Health Law n ° 2004-806 of August 9, 2004, you would be entitled to receive compensation within the framework of this insurance contract.

This research received the favourable opinion of the Committee for the Protection of Persons South East VI at the meeting of .............................. As well as the prior authorization of the competent health authority dated .......................... .. This research may be interrupted, if the circumstances so require, by the sponsor or at the request of the competent authority.

**Data privacy:**

In the framework of the biomedical research to which the CHU of Clermont-Ferrand proposes to participate, a computerized treatment of your personal data will be implemented in order to analyse the results of the research with respect to the objective of this research was presented to you.
Information about the study collected by the investigator is treated confidentially.
In accordance with the Data Protection and Freedom Law, your name is automatically replaced by a code number whose correspondence is known only to the investigating physicians.

The data will be the subject of an anonymous computerized treatment and their consultation will be authorized to the collaborators participating in the research, designated by the promoter and possibly to the representative of the health authorities.
In accordance with the provisions of the law on data processing, files and freedoms, you have a right of access and rectification to the doctor who follows you in the course of research.
You also have the right to object to the transmission of data subject to professional secrecy that may be used in the course of this research and to be processed.
You can also access directly or through a doctor of your choice all your medical data in accordance with the provisions of Article L. 1111-7 of the Public Health Code. These rights are exercised with the doctor who follows you in the course of research and who knows your identity.
You are free to accept or refuse to participate in this research. In addition, you may exercise your right to withdraw from this search at any time.
In addition, you will be kept informed of the overall results of this research at the end of the study.

If you wish, you can at any time request additional information about the study from the medical staff of the gastroenterology department of Clermont-Ferrand at .............................. Or .......... .................... from Monday to Friday from 8 am to 5 pm.
In the event of an emergency and throughout the duration of the study, you can reach the medical team that follows you as part of your participation in this study at the following N ° ....................... . Or, failing that, the physician of astreinte service of gastroenterology of Clermont-Ferrand at .................................. ..
When you have read this briefing note and obtained the answers to the questions you have asked by asking the investigating physician, you will be offered, if you agree, to give your written consent by signing the document prepared at that effect.

Date: ...... / ...... / ......

Signature of Physician

Signature of Subject
Preceded by the words "Read and understood

**Assessment of the Effectiveness and Safety of Ethosuximide in the Treatment of Abdominal Pain related to Irritable Bowel Syndrome**

**Sponsor**

University Hospital of Clermont-Ferrand
58 Rue de Montalembert,
63000 Clermont-Ferrand, France

**Principal investigator**

Pr Michel DAPOIGNY
Gastroenterology service
University Hospital Estaing
63000 Clermont-Ferrand, France

I undersigned M. (surname, first name) ...................................... Born on _ _ / _ _/ _ _ _ _
Remaining .........................................................................................
Declares: that the Doctor (surname, first name, telephone) ........................................ ............................................... Proposed to me to participate in the above-mentioned study, that he explained to me in detail the protocol, which he made known to me in particular:

- The objective, method and duration of the study

- Potential constraints and risks

- My right to refuse to participate and withdraw my consent at any time without having to justify myself

- My registration requirement a social security regimen

- That, if I wish, at the end, I would be informed by the investigating doctor of his overall results

- That I will not be allowed to participate in any other clinical studies during the total duration of the study (16 weeks ± 1 week).

- that the Committee for the Protection of Persons South East VI has issued a favourable opinion on ......... ............, as well as the prior authorization of the competent health authority dated ... ........................

- That within the framework of this study the promoter, the CHU of Clermont-Ferrand, has taken out insurance covering this research.

- The exclusion period defined in this study is 30 days, during which time you will not be able to participate in another clinical research protocol.

Information about the study collected by the investigator is treated confidentially.

I agree that these data may be the subject of an anonymous computerized treatment. I have noticed that the right of access provided by the law of 6 August 2004 relating to data processing, files and freedoms is exercised at all times with the doctor who follows me in the course of the research and who knows my identity. I may exercise my right to rectify and object to the same doctor who will contact the research sponsor.

I accept my registration in the National File of persons who lend themselves to biomedical research (Article L 1121-16 of the Code of Public Health).

After freely discussing and answering all of my questions, I agree freely and voluntarily to participate in this biomedical research under the conditions specified in the information and consent form.

Name and first name of the subject: .................................. ..
Date: ....... / ....... / .......
Signature preceded by the words "Read and understood":

Name of Principal Investigator: ....................
Date: ....... / ....... / .......
Signature

APPENDICE 2 – GENETIC STUDY FORM

**PARTICIPATION OF CONSENT FORM TO BIOMEDICAL RESEARCH - GENETIC STRAND**

Title: *Assessment of the Effectiveness and Safety of Ethosuximide in the Treatment of Abdominal Pain related to Irritable Bowel Syndrome*

**Sponsor**
C.H.U. de Clermont-Ferrand
58 Rue de Montalembert
63003 Clermont-Ferrand, FRANCE

**Coordinating investigator** :
Service de gastroentérologie,
C.H.U. Estaing,
63000 Clermont-Ferrand, France

During this study, a blood test (5 ml) will be performed by a nurse during your first visit to the gastroenterology department for genetic analysis. This blood sampling will be carried out with a view to the constitution of a biological collection which will be analysed later, in order to possibly establish a correlation between genetics, efficacy and undesirable effects of the treatment tested in the IBSET study.

This sampling will allow a posteriori to carry out a genetic analysis on the detection and the identification of the variants of the genes of the cytochrome P450 3A4 and 2E1 involved in the hepatic metabolism of the ethosuximide. These genes encode proteins present on the surface of the body's cells that have an impact on the fate of drugs in your body (the way your body transforms and eliminates drugs), and can have an impact on the response to treatment (efficacy, severity of adverse effects ...).

These dosages are intended to improve the knowledge and management of any adverse effects induced by the treatment. As for all other samples taken from the study, these samples will be treated anonymously and identified using a code.

All samples from the study will be stored in the gastroenterology department and the University Hospital of Clermont-Ferrand in optimal conditions temperature monitoring for further analysis, only as part of this study. After analysis and after the end of the study, these samples will be destroyed.

In order to validate your agreement or disagreement, please complete the information below:

I, the undersigned M ............................................ ................................. (Last name First Name),

Born on _ _ / _ _ / _ _ _ _ at .................................. (City of birth)

Residing: ........................................................................................................

Authorizes / does not authorize (delete the unnecessary mention) the taking of a blood sample for the genetic assay that will detect and identify the presence of variants of the CYP3A4 and CYP2E1 genes involved in the metabolism of ethosuximide.

I received a copy of this document and I was informed that a copy would also be retained by the investigator in conditions guaranteeing privacy and I consent.

Name and first name of the investigator:

…………………………………………………

Date :……./……./…….

AT : ……………………………

Signature

First and last name of the subject:

…………………………………………………

Date :……./……./…….

AT : ……………………………

Signature *preceded by the words "Read and understood"*

APPENDICE 3 – BIOLOGICAL SPECIMEN

| **Blood Test** | **Time** |
|---|---|
| ALT | D0-7 (screening) |
| AST | D0-7 (screening) |
| ALP | D0-7 (screening) |
| GGT | D0-7 (screening) |
| Creatininemia | D0-7 (screening) |
| Haematocrit | D0-7 (screening) |
| Beta-HCG | D0-7 (screening) |
| Genetic analysis | D0-7 (screening) |

APPENDICE 3 – REFRENCES


1. Thompson, W. G. Irritable bowel syndrome: a management strategy. *Baillieres Best Pract Res Clin Gastroenterol* **13**, 453–460 (1999).
2. Drossman, D. A. *et al.* Severity in irritable bowel syndrome: a Rome Foundation Working Team report. *Am. J. Gastroenterol.* **106**, 1749–1759; quiz 1760 (2011).
3. Müller-Lissner, S. A. *et al.* Epidemiological aspects of irritable bowel syndrome in Europe and North America. *Digestion* **64**, 200–204 (2001).
4. Dapoigny, M. *et al.* Irritable bowel syndrome in France: a common, debilitating and costly disorder. *Eur J Gastroenterol Hepatol* **16**, 995–1001 (2004).
5. Mönnikes, H. Quality of life in patients with irritable bowel syndrome. *J. Clin. Gastroenterol.* **45 Suppl**, S98-101 (2011).
6. Longstreth, G. F. *et al.* Functional bowel disorders. *Gastroenterology* **130**, 1480–1491 (2006).
7. Piche, T. *et al.* Impact of functional bowel symptoms on quality of life and fatigue in quiescent Crohn disease and irritable bowel syndrome. *Neurogastroenterol. Motil.* **22**, 626-e174 (2010).
8. Brun-Strang, C., Dapoigny, M., Lafuma, A., Wainsten, J. P. & Fagnani, F. Irritable bowel syndrome in France: quality of life, medical management, and costs: the Encoli study. *Eur J Gastroenterol Hepatol* **19**, 1097–1103 (2007).
9. Piche, T. *et al.* Mast cells and cellularity of the colonic mucosa correlated with fatigue and depression in irritable bowel syndrome. *Gut* **57**, 468–473 (2008).
10. Piche, T. *et al.* Impaired intestinal barrier integrity in the colon of patients with irritable bowel syndrome: involvement of soluble mediators. *Gut* **58**, 196–201 (2009).
11. Ritchie, J. Pain from distension of the pelvic colon by inflating a balloon in the irritable colon syndrome. *Gut* **14**, 125–132 (1973).
12. Whitehead, W. E. Patient subgroups in irritable bowel syndrome that can be defined by symptom evaluation and physical examination. *Am. J. Med.* **107**, 33S-40S (1999).
13. Azpiroz, F. *et al.* Mechanisms of hypersensitivity in IBS and functional disorders. *Neurogastroenterol. Motil.* **19**, 62–88 (2007).
14. Akbar, A. *et al.* Expression of the TRPV1 receptor differs in quiescent inflammatory bowel disease with or without abdominal pain. *Gut* **59**, 767–774 (2010).
15. Cervero, F. & Laird, J. M. A. Role of ion channels in mechanisms controlling gastrointestinal pain pathways. *Curr Opin Pharmacol* **3**, 608–612 (2003).
16. Wood, J. D. Neuropathy in the brain-in-the-gut. *Eur J Gastroenterol Hepatol* **12**, 597–600 (2000).
17. Todorovic, S. M. & Jevtovic-Todorovic, V. The role of T-type calcium channels in peripheral and central pain processing. *CNS Neurol Disord Drug Targets* **5**, 639–653 (2006).
18. Sekiguchi, F. & Kawabata, A. T-type Calcium Channels: Functional Regulation and Implication in Pain Signaling. *J. Pharmacol. Sci.* **122**, 244–250 (2013).
19. Scanzi, J. *et al.* Colonic overexpression of the T-type calcium channel Cav 3.2 in a mouse model of visceral hypersensitivity and in irritable bowel syndrome patients. *Neurogastroenterol. Motil.* (2016) doi:10.1111/nmo.12860.
20. Marger, F. *et al.* T-type calcium channels contribute to colonic hypersensitivity in a rat model of irritable bowel syndrome. *Proc. Natl. Acad. Sci. U.S.A.* **108**, 11268–11273 (2011).
21. Todorovic, S. M. & Jevtovic-Todorovic, V. Neuropathic pain: role for presynaptic T-type channels in nociceptive signaling. *Pflugers Arch.* **465**, 921–927 (2013).
22. Hamidi, G. A. *et al.* Ethosuximide reduces allodynia and hyperalgesia and potentiates morphine effects in the chronic constriction injury model of neuropathic pain. *Eur. J. Pharmacol.* **674**, 260–264 (2012).
23. Dogrul, A. *et al.* Reversal of experimental neuropathic pain by T-type calcium channel blockers. *Pain* **105**, 159–168 (2003).
24. Matthews, E. & Dickenson, A. Effects of ethosuximide, a T-type Ca(2+) channel blocker, on dorsal horn neuronal responses in rats. *European journal of pharmacology* **European journal of pharmacology**, 141–9 (2001).
25. Okubo, K. *et al.* Inhibition of T-type calcium channels and hydrogen sulfide-forming enzyme reverses paclitaxel-evoked neuropathic hyperalgesia in rats. *Neuroscience* **188**, 148–156 (2011).

26. Flatters, S. J. L. & Bennett, G. J. Ethosuximide reverses paclitaxel- and vincristine-induced painful peripheral neuropathy. *Pain* **109**, 150–161 (2004).
27. Munro, G., Erichsen, H. K. & Mirza, N. R. Pharmacological comparison of anticonvulsant drugs in animal models of persistent pain and anxiety. *Neuropharmacology* **53**, 609–618 (2007).
28. Kawabata, A. *et al.* Hydrogen sulfide as a novel nociceptive messenger. *Pain* **Pain**, 74–81 (2007).
29. Barton, M., Eberle, E. & Shannon, H. The antihyperalgesic effects of the T-type calcium channel blockers ethosuximide, trimethadione, and mibefradil. *European journal of pharmacology* **European journal of pharmacology**, 79–85 (2005).
30. Shannon, H. E., Eberle, E. L. & Peters, S. C. Comparison of the effects of anticonvulsant drugs with diverse mechanisms of action in the formalin test in rats. *Neuropharmacology* **48**, 1012–1020 (2005).
31. Chen, W.-K. *et al.* Ca(v)3.2 T-type Ca2+ channel-dependent activation of ERK in paraventricular thalamus modulates acid-induced chronic muscle pain. *The Journal of neuroscience : the official journal of the Society for Neuroscience* **The Journal of neuroscience : the official journal of the Society for Neuroscience**, 10360–8 (2010).
32. Todorovic, S. M., Rastogi, A. J. & Jevtovic-Todorovic, V. Potent analgesic effects of anticonvulsants on peripheral thermal nociception in rats. *Br. J. Pharmacol.* **140**, 255–260 (2003).
33. Matsunami, M., Kirishi, S., Okui, T. & Kawabata, A. Hydrogen sulfide-induced colonic mucosal cytoprotection involves T-type calcium channel-dependent neuronal excitation in rats. *J. Physiol. Pharmacol.* **63**, 61–68 (2012).
34. Sekiguchi, F. *et al.* AKAP-dependent sensitization of Ca(v) 3.2 channels via the EP (4) receptor/cyclic AMP pathway mediates prostaglandin E (2) -induced mechanical hyperalgesia. *Br. J. Pharmacol.* (2012) doi:10.1111/j.1476-5381.2012.02174.x.
35. Xu, G.-Y. *et al.* The endogenous hydrogen sulfide producing enzyme cystathionine-beta synthase contributes to visceral hypersensitivity in a rat model of irritable bowel syndrome. *Mol Pain* **5**, 44 (2009).
36. Li, L. *et al.* Upregulation of cystathionine beta-synthetase expression by nuclear factor-kappa B activation contributes to visceral hypersensitivity in adult rats with neonatal maternal deprivation. *Mol Pain* **8**, 89 (2012).
37. Maeda, Y. *et al.* Hyperalgesia induced by spinal and peripheral hydrogen sulfide: evidence for involvement of Cav3.2 T-type calcium channels. *Pain* **142**, 127–132 (2009).
38. Sekiguchi, F. *et al.* Endogenous and exogenous hydrogen sulfide facilitates T-type calcium channel currents in Cav3.2-expressing HEK293 cells. *Biochem. Biophys. Res. Commun.* **445**, 225–229 (2014).
39. Müller-Lissner, S. *et al.* Subject's Global Assessment of Relief: an appropriate method to assess the impact of treatment on irritable bowel syndrome-related symptoms in clinical trials. *J Clin Epidemiol* **56**, 310–316 (2003).
40. Eypasch, E. *et al.* Gastrointestinal Quality of Life Index: development, validation and application of a new instrument. *Br J Surg* **82**, 216–222 (1995).
41. Bushnell, D. M., Martin, M. L., Ricci, J.-F. & Bracco, A. Performance of the EQ-5D in patients with irritable bowel syndrome. *Value Health* **9**, 90–97 (2006).
42. Francis, C. Y., Morris, J. & Whorwell, P. J. The irritable bowel severity scoring system: a simple method of monitoring irritable bowel syndrome and its progress. *Aliment. Pharmacol. Ther.* **11**, 395–402 (1997).
43. Ford, A. C. *et al.* Effect of antidepressants and psychological therapies, including hypnotherapy, in irritable bowel syndrome: systematic review and meta-analysis. *Am. J. Gastroenterol.* **109**, 1350–1365; quiz 1366 (2014).

**Statistical Analysis Plan**

Evaluation of the efficacy of ethosuximide in the treatment of abdominal pain associated with irritable bowel syndrome

EudraCT identifying number: 2016-002110-42

Date: 23/03/2022


Study sponsor
University Hospital of Clermont-Ferrand
58 Rue de Montalembert
63003 Clermont-Ferrand Cedex 1

Principal coordinator
Julien SCANZI, MD, PhD
Dept. of gastroenterology
Estaing Hospital, University Hospital of Clermont-Ferrand
Tel: +33 (0)4 73 75 05 28
Email: jscanzi@chu-clermontferrand.fr

Study methodology
Bruno PEREIRA, PhD
Biostatistic unit, Delegation Recherche Clinique & Innovation (DRCI), University Hospital of Clermont-Ferrand
58, Rue Montalembert 63003 Clermont-Ferrand cedex
Tel: +33 (0) 473754964
Email: bpereira@chu-clermontferrand.fr

## 1. Trial summary

### 1.1 Primary objective

To evaluate the efficacy of ethosuximide versus placebo, administered in add-on therapy, on intensity of abdominal pain related to IBS and to evaluate the score of the Subject Global Assessment of Relief (SGA scale) after 12 weeks of treatment.

### 1.2 Secondary objectives

The secondary objectives concern the effects of ethosuximide on:

►the severity of IBS symptoms
►the quality of life (physical and mental)
►the safety of ethosuximide.

The study discontinuation rate and analgesics and transit regulator consumption were assessed throughout the study.

### 1.3 Trial design

Multicentre, national, randomised, double-blinded, placebo-controlled, parallel group clinical trial with two parallel groups:

- Group A: ethosuximide
- Group B: placebo

### 1.4 Randomization

After patient informed consent was obtained and eligible criteria respected, enrolled patients were randomly allocated to receive either ethosuximide or placebo in a 1:1 ratio. Randomisation was conducted over a dedicated, password-protected, SSL-encrypted website (CSOnline, Clinsight) to allow concealed allocation. Each patient was given a unique patient number and randomisation number.

Computer-generated random numbers was used to generate allocation sequence with stratification by centre and a 6-block random sequence. Sequentially numbered containers were used to implement the random allocation sequence.

### 1.5 Blinding

Participants were given blinded medication. Trial medication (syrup of ethosuximide or placebo) were visually identical and packaged into indistinguishable bottle by the pharmacist of the coordinating centre and delivered to each specific study site. Only the pharmacy of the study coordinating centre was aware of the bottle composition (coding list). The receipt, storage and dispensing of the blinded trial medication were conducted by the pharmacy department in each individual study site.

The allocation of trial medication was determined by the web-based randomisation system. Each study site had sufficient bottles to be allocated to patients included.

The allocated trial medication was blinded to the patient, the clinical staff caring for the patient, the investigators, the outcome assessors, the data manager, and the statistician conducting the analyses. At each participating centre, data were collected and entered into the web-based electronic case report form (eCRF) by trial or clinical trained personal (clinical research associate), blinded to the allocation group, under the supervision of the trial site investigators.

### 1.6 Eligibility criteria

#### 1.6.1 Inclusion criteria

► Man or woman aged 18 years or more.
► Negative pregnancy test and effective contraception.
► IBS defined by ROME IV criteria.

► Treatment failure (11-point NRS abdominal pain ≥ 4/10) for at least 3 months despite stable treatment for a month, if any.
► Patients affiliated to the regime of the French Social Security.
► Patients able to deliver a free and informed consent.

#### 1.6.2 Non-inclusion criteria

► Breastfeeding.
► Diabetic patients.
► Chronic pain of greater intensity than the pain related to IBS.
► Renal or hepatic impairment defined by significant liver function (liver enzymes >3 × normal values, cholestasis) and renal (modification of the diet in renal disease (MDRD) <60 mL/min) abnormalities.
► Current or history of severe depression (hospitalisation, long-term antidepressant treatment).
► Addiction to alcohol and/or drugs.
► Patient already treated with antiepileptic.
► Epilepsy.
► Allergy to succinimide (ethosuximide, methsuximide, phensuximide).
► Psychotic disorders.
► Insufficient cooperation and understanding to adhere strictly to the conditions provided by the study.
► Patients undergoing a measure of legal protection.

### 1.7 Sample size

According to previous work presented in literature, we have estimated that a sample size of n=130 patients per randomised group, for a two-sided type I error at 5%, would provide 90% statistical power of detecting an absolute difference of 20% between ethosuximide and placebo in the primary outcome. Based on the meta-analysis of Ford et al, the estimated median responder rate in the placebo group was 35%. A slightly higher rate than the previously mentioned meta-analysis (32% of responders under drug therapies), in order to avoid underestimating the placebo effect. Finally, a total of n=290 patients (145 by group) was considered to take into account lost to follow-up (10%). A blinded interim analysis was planned after enrolment of the first 100 patients using the Lan and DeMets rule (East software, Cytel, Cambridge, Massachusetts, USA) to study safety of study treatment (ethosuximide and placebo) and efficacy, to continue or stop the study in case of futility and to recalculate the number of patients needed. The type I error is fixed at 0.03 for this interim analysis.

### 1.8 Primary outcome measure

The primary outcome was the rate of responders to treatment: a patient will be considered as a responder if he satisfies two conditions: (1) a decrease of at least 30% of the score of abdominal pain (11-point NRS) compared with the score before treatment, and (2) a significant global improvement defined by a score of 4 or 5 on the SGA scale.

### 1.9 Secondary outcome measures

Secondary outcome measures include:

- The quality of life was evaluated by the GIQLI and EQ-5D-3L (EuroQol group), which assess the physical and mental health of IBS patient.
- The severity of IBS symptoms was evaluated by IBS-SSS questionnaire and Bristol scale.
- The safety of ethosuximide was evaluated by daily collection of adverse effect by patient.
- Analgesic consumption throughout the study.

## 2. Analysis plan

### 2.1 Generality

All analyses will be performed with the use of Stata software (version 15, StataCorp, College Station, USA) before the breaking of the randomization code, according to International Conference on Harmonization-Good Clinical Practice guidelines.

The primary analysis will be conducted in the intention-to-treat (ITT), modified intention-to-treat (mITT) and per-protocol populations. We will perform a per-protocol analysis on the primary outcome. The criteria for including patients in the modified ITT and in the per-protocol populations, respectively, are provided below.

Baseline variables will be reported as numbers and percentages for categorical variables and medians with interquartile ranges (IQRs) for continuous variables. As according to the CONSORT 2010 statement, group differences in baseline variables will not be compared using significance testing unless specifically requested by peer reviewers.

### 2.2 Study population

<u>Intention-to treat (ITT) population</u>: All randomised patients.
Because the study is a proof of concept, this population will not be analysed in the IBSET study.

<u>Modified intention-to-treat population (mITT)</u>: All randomised patients except patients who:

- Patients who withdrawn consent for the use of data

OR

- Patients who did not have any of the interventions

OR

- Patients who did not be eligible for randomisation according to inclusion/non-inclusion criteria (screen fail or absence of baseline pain intensity [primary outcome])

OR

- Patients with major deviation (primary outcome not available, non-compliance)

<u>Per-protocol population</u>: All randomised patients except patients having one or more major protocol violations defined as:

- Patients who accidentally received the wrong treatment

OR

- Patients who discontinued study treatment

OR

- Patients who did not have any of the interventions

OR

- Patients who withdrawn consent for the use of data

OR

- Patients who did not be eligible for randomisation according to inclusion/non-inclusion criteria (screen fail or absence of baseline pain intensity [primary outcomes])

OR

- Patients with major deviation (primary outcome not available, non-compliance)

### 2.3 Primary analysis

The rate of responders to treatment was reported for each treatment group. The primary effect estimate was the relative risk of response, reported with 95% confidence intervals (CI). The absolute difference and 95% CI was also reported. The rate of responders to treatment was compared between the treatment groups using unadjusted chi-squared test (or Fisher's exact test, as appropriate).

### 2.4 Secondary analyses

Multiple logistic mixed regression was used to identify relevant covariates in the mITT population anticipated with the primary outcome (criterion for entering variables was $P<0.10$), in addition to the stratification variables (centre):

- Categorical covariates
  - Gender (M/F)
  - Type of IBS (diarrhoea, constipation and mixed)
  - Ongoing analgesic treatments for IBS at inclusion (Y/N)

- Continuous covariates
  - Age (years)
  - IBS Duration (months)
  - Pain intensity (treated as continuous variable and then categorized as ordinal covariate ≤3/10, 4-6/10, ≥7/10).

Adjusted analyses was performed with the use of robust random-effect Poisson generalized linear mixed model regression with robust variance for binary outcomes, with centre as a random effect. Particular attention was paid to multicollinearity using the Farrar-Glauber test and variance inflation factor (VIF). The Akaike information criterion and Bayesian information criterion were calculated and used as model diagnostics to determine how well the model fit improved following addition of covariates. Significance of the intervention was assessed based on p-values and risk ratios with 95% CIs calculated from this regression.

Dichotomous secondary endpoints were compared using chi-squared test. Continuous variables were presented as mean and standard deviations (as median and quartiles, otherwise) and were compared with the use of the unpaired *t* test or the Mann-Whitney U test when appropriate (the Shapiro-Wilk test was used to assess normality, and the Fisher-Snedecor test to assess homoscedasticity). Adjusted analyses were performed with the use of robust random-effect Poisson generalized linear mixed model regression with robust variance for binary outcomes, multinomial logistic mixed model for categorical outcome, and linear mixed regression for continuous outcomes, with site as a random effect. Results were presented as absolute differences and risk ratios with CIs for binary and categorical data and between-group difference with CIs for continuous data.

Longitudinal analyses concerning repeated measures were studied using random-effect models (linear or generalized linear), to take into account patient as random-effect (slope and intercept), nestled in center random-effect.

Planned subgroup analyses were assess heterogeneity of the effect of ethosuximide on the primary outcome measure in pre-specified subgroups of patients with IBS subgroup and gender. According to usual recommendations, the interactions between group randomization and subgroups was evaluated in regression models (i.e., random-effects models, linear or generalized linear).

A descriptive analysis of stopping and discontinuations in view of dose escalation was considered. A particular focus was done to safety and lose to follow-up. A study of abandonment considered as censored data was proposed using the Kaplan-Meier estimation. The proportional-hazard hypothesis was verified using Schoenfeld's test and plotting residuals.
Finally, a sensitivity analysis was performed and the nature of missing data was studied (missing at random or not). According to this, the most appropriate approach to the imputation of missing data was proposed (MICE, spline interpolation, LOCF, BOCF…).

A two-sided P value of less than 0.05 was considered for statistical significance of all analyzes. The Hochberg procedure was used to adjust for multiple testing of each component of the composite primary outcome ((1) decrease of at least 30% of the intensity of abdominal pain (11-point NRS) compared with the score before treatment and (2) significant global improvement defined by a score of 4 or 5 on the SGA scale). Because of the potential for type 1 error due to multiple comparisons, findings from analyses of secondary endpoints were interpreted as exploratory.
