# Supplementary material for: Ethosuximide and Irritable Bowel Syndrome–Related Abdominal Pain: A Randomized Clinical Trial
Source: JAMA Netw Open. 2026 Jan 8;9(1):e2551368. doi: 10.1001/jamanetworkopen.2025.51368 (PMC12784227; doi:10.1001/jamanetworkopen.2025.51368)
Supplement: Supplement 2. — eFigure. Forest plot of relative risk comparing responder status with patient characteristics and study treatments eTable 1. Secondary efficacy end points: ITT population eTable 2. Secondary efficacy end points: PP population [file jamanetwopen-e2551368-s002.pdf]

Supplemental Online Content

Kerckhove N, Zerbib F, Chambaz M, et al; for the IBSET Investigator Group (IIG) . Ethosuximide and irritable bowel syndrome–related abdominal pain: a randomized clinical trial. *JAMA Netw Open*. 2026;9(1):e2551368. doi:10.1001/jamanetworkopen.2025.51368

eFigure 1. Forest plot of relative risk comparing responder status with patient characteristics and study treatments

eTable 1. Secondary efficacy end points: ITT population

eTable 2. Secondary efficacy end points: PP population

This supplemental material has been provided by the authors to give readers additional information about their work.

© 2026 Kerckhove N et al. *JAMA Network Open*.

Supplementary Table 1

Questionnaires	Ethosuximide Group n = 64	Placebo Group N = 60	Treatment effect
**SGA+ (score ≥ 4/5) – % [95%CI] mean ± SD and relative risk [95%CI]**			
**Week 4 (51/53)**	14.1% [8.1%-31.0%]	23.3% [12.9%-36.1%]	0.60 [0.28-1.29]
**Week 8 (35/51)**	17.2% [7.9%-26.4%]	18.3% [8.5%-28.1%]	0.94 [0.44-2.00]
**Week 12 (45/51)**	25.0% [14.4%-35.6%]	26.7% [15.5%-37.9%]	0.94 [0.52-1.07]
**SGA score (/5) – mean ± SD and mean difference [95%CI]**			
**Week 4 (51/53)**	2.5 ± 1.1	2.9 ± 0.9	-0.49 [-0.89; 0.10]
**Week 8 (35/51)**	2.6 ± 1.1	2.8 ± 0.9	-0.23 [-0.61; 0.16]
**Week 12 (45/51)**	2.7 ± 1.2	2.8 ± 1.0	-0.20 [-0.61; 0.21]
**Patients with abdominal pain intensity variation at least -30% (vs. baseline) – % [95%CI] and relative risk [95%CI]**			
**Week 4 (51/55)**	17.2% [7.9%-26.4%]	31.7% [19.9%-43.4%]	0.54 [0.28-1.04]
**Week 8 (36/49)**	15.6% [6.7%-24.5%]	25.0% [14.0%-36.0%]	0.62 [0.30-1.28]
**Week 12 (41/51)**	26.6% [15.7%-37.4%]	36.7% [24.5%-48.9%]	0.72 [0.43-1.23]
**Abdominal pain intensity - NRS score (0-10) – mean ± SD and mean difference [95%CI]**			
**Baseline (64/60)**	6.1 ± 1.2	6.0 ± 1.5	/
**Week 4 (51/55)**	5.4 ± 2.0	5.0 ± 2.3	0.28 [-0.26; 0.82]
**Week 8 (36/49)**	5.3 ± 2.1	5.0 ± 2.4	0.24 [-0.30; 0.78]
**Week 12 (41/51)**	5.1 ± 2.2	4.7 ± 2.7	0.26 [-0.28; 0.80]
**GIQLI total score– mean ± SD and mean difference [95%CI]**			
**Baseline (63/57)**	66.8 ± 13.0	69.5 ± 15.5	/
**Week 4 (51/51)**	65.9 ± 17.1	65.8 ± 17.1	2.56 [-3.81; 8.92]
**Week 8 (33/51)**	64.2 ± 21.1	65.1 ± 17.1	1.46 [-4.90; 7.82]
**Week 12 (40/49)**	62.4 ± 26.1	63.8 ± 20.1	1.01 [-5.35; 7.37]
**IBS-SSS total score – mean ± SD and mean difference [95%CI]**			
**Baseline (63/59)**	355.2 ± 70.9	364.98 ± 61.5	/
**Week 4 (51/53)**	303.3 ± 106.1	344.48 ± 87.3	31.44 [3.49; 59.39]
**Week 8 (33/51)**	296.3 ± 112.5	335.47 ± 92.2	29.45 [1.51; 57.40]
**Week 12 (40/49)**	277.1 ± 128.8	327.44 ± 102.7	40.55 [12.60; 68.49]
**EQ-5D-3L scores – mean ± SD and mean difference [95%CI]**			
**Total score - Baseline (63/59)**	0.57 ± 0.23	0.59 ± 0.24	/
**Total score – Week 4 (51/53)**	0.64 ± 0.24	0.68 ± 0.21	-0.02 [-0.08; 0.05]
**Total score – Week 8 (34/50)**	0.62 ± 0.25	0.65 ± 0.24	-0.01 [-0.07; 0.05]
**Total score – Week 12 (41/47)**	0.62 ± 0.26	0.69 ± 0.26	-0.05 [-0.11; 0.01]
**HRQoL score baseline (63/59)**	49.0 ± 21.7	45.9 ± 15.4	/
**HRQoL score Week 4 (51/53)**	52.7 ± 22.4	45.5 ± 20.4	-4.50 [-11.13; 2.12]
**HRQoL score Week 8 (34/50)**	50.2 ± 22.3	49.2 ± 22.6	1.67 [-4.95; 8.30]
**HRQoL score Week 12 (41/47)**	51.4 ± 25.2	48.8 ± 21.3	0.19 [-6.44; 6.81]

Mean differences were estimated using linear mixed models with a patient-level random effect (except for SGA score calculated at each time point in the absence of baseline values). They represent the difference in the change from baseline to follow-up between the randomization groups (time × group interaction).

Intra-class-correlation coefficient for random-effect (p-values for variance components of patient random-effect): 0.73 (p<0.001) for abdominal pain intensity, 0.67 (p<0.001) for GIQLI total score, 0.54 (p<0.001) for IBS-SSS total score, 0.73 (p<0.001) for Total EQ-5D-3L score and 0.63 (p<0.001) for HRQoL score.

© 2026 Kerckhove N et al. *JAMA Network Open*.

Supplementary Table 2

Questionnaires	Ethosuximide Group N = 28	Placebo Group N = 41	Treatment effect
**SGA+ (score ≥ 4/5) – % [95%CI] and relative risk [95%CI]**			
**Week 4 (28/39)**	25.0% [11.4%-45.2%]	34.1% [20.6%-50.7%]	0.73 [0.29-1.84]
**Week 8 (27/40)**	39.3% [22.1%-59.3%]	22.0% [11.1%-38.0%]	1.79 [0.71-4.51]
**Week 12 (28/41)**	60.7% [40.7%-77.9%]	29.3% [16.6%-45.7%]	**2.07 [1.82-5.23]**
**SGA score (/5) – mean ± SD and mean difference [95%CI]**			
**Week 4 (28/39)**	2.9 ± 1.0	3.1 ± 1.0	-0.19 [-0.67; 0.29]
**Week 8 (27/40)**	3.2 ± 1.0	2.9 ± 0.9	0.30 [-0.15; 0.75]
**Week 12 (28/41)**	3.4 ± 1.2	2.9 ± 1.0	0.55 [-0.02; 1.08]
**Patients with abdominal pain intensity variation at least -30% (vs. baseline) – % [95%CI] and relative risk [95%CI]**			
**Week 4 (28/41)**	32.1% [14.8%-49.4%]	41.5% [26.4%-56.6%]	0.77 [0.31-1.95]
**Week 8 (28/41)**	32.1% [14.8%-49.4%]	34.1% [19.6%-48.6%]	0.94 [0.37-2.37]
**Week 12 (28/41)**	57.1% [38.8%-75.4%]	41.5% [26.4%-56.6%]	1.38 [0.55-3.47]
**Abdominal pain intensity - NRS score (0-10) – mean ± SD and mean difference [95%CI]**			
**Baseline (28/41)**	5.7 ± 1.2	5.9 ± 1.2	/
**Week4 (28/41)**	4.4 ± 2.2	4.8 ± 2.2	-0.19 [-1.04; 0.65]
**Week8 (28/41)**	4.1 ± 2.2	4.7 ± 2.2	-0.45 [-1.30; 0.40]
**Week12 (28/41)**	3.6 ± 2.1	4.6 ± 2.5	-0.82 [-1.66; 0.03]
**QIQLI total score – mean ± SD and mean difference [95%CI]**			
**Baseline (28/41)**	67.8 ± 11.4	72.0 ± 12.3	/
**Week4 (28/38)**	71.7 ± 13.5	69.3 ± 15.6	6.59 [-1.25; 14.43]
**Week8 (27/40)**	69.5 ± 19.5	67.9 ± 15.9	5.73 [-2.11; 13.57]
**Week12 (28/39)**	74.3 ± 17.7	68.8 ± 13.9	9.68 [1.84; 17.52]
**IBS-SSS total score – mean ± SD and mean difference [95%CI]**			
**Baseline (28/41)**	351.6 ± 75.0	341.6 ± 77.3	/
**Week4 (28/38)**	302.3 ± 99.4	292.9 ± 103.9	-0.59 [-49.09; 47.92]
**Week8 (27/40)**	270.9 ± 99.9	281.0 ± 117.6	-20.16 [-68.67; 28.34]
**Week12 (28/39)**	257.1 ± 107.0	273.2 ± 118.7	-26.17 [-74.67; 22.33]
**EQ-5D-3L scores – mean ± SD and mean difference [95%CI]**			
**Total score - Baseline (28/41)**	0.64 ± 0.16	0.68 ± 0.17	/
**Total score – Week 4 (28/38)**	0.78 ± 0.14	0.75 ± 0.15	0.08 [-0.01; 0.16]
**Total score – Week 8 (27/40)**	0.76 ± 0.17	0.74 ± 0.16	0.07 [-0.02; 0.15]
**Total score – Week 12 (28/39)**	0.74 ± 0.18	0.77 ± 0.17	0.02 [-0.06; 0.10]
**HRQoL score - baseline (28/41)**	48.7 ± 17.5	54.1 ± 19.6	/
**HRQoL score – Week 4 (28/38)**	50.5 ± 21.3	53.2 ± 20.1	2.76 [-7.55; 13.07]
**HRQoL score – Week 8 (27/40)**	60.7 ± 24.4	56.5 ± 21.7	9.65 [-0.67; 19.96]
**HRQoL score – Week 12 (28/39)**	61.8 ± 21.0	57.6 ± 24.6	9.61 [-0.70; 19.92]

Mean differences were estimated using linear mixed models with a patient-level random effect (except for SGA score calculated at each time point in the absence of baseline values). They represent the difference in the change from baseline to follow-up between the randomization groups (time × group interaction).
Intra-class-correlation coefficient for random-effect (p-values for variance components of patient random-effect): 0.61 (p<0.001) for abdominal pain intensity, 0.40 (p<0.001) for GIQLI total score, 0.50 (p<0.001) for IBS-SSS total score, 0.47 (p<0.001) for Total EQ-5D-3L score and 0.48 (p<0.001) for HRQoL score.

© 2026 Kerckhove N et al. *JAMA Network Open*.

Supplementary Figure 1

Items	Relative Risk	[95% Conf. Interval]
Ethosuximide (vs. placebo) - mITT	1.62	[0.80;3.27]
**Ethosuximide (vs. placebo) - PP**	**2.15**	**[1.14;4.04]**
Female (vs. Male) - mITT	0.96	[0.48;1.91]
Female (vs. Male) - PP	0.65	[0.33;1.27]
Age of patient - mITT	1.01	[0.99;1.03]
Age of patient - PP	1.02	[1.00;1.04]
IBS type D (vs. type C) - mITT	1.89	[0.72;4.96]
IBS type D (vs. type C) - PP	1.52	[0.65;3.60]
IBS type M (vs. type C) - mITT	1.95	[0.81;4.67]
IBS type M (vs. type C) - PP	2.04	[0.94;4.42]
IBS duration - mITT	1.00	[0.97;1.04]
IBS duration - PP	1.00	[0.97;1.03]

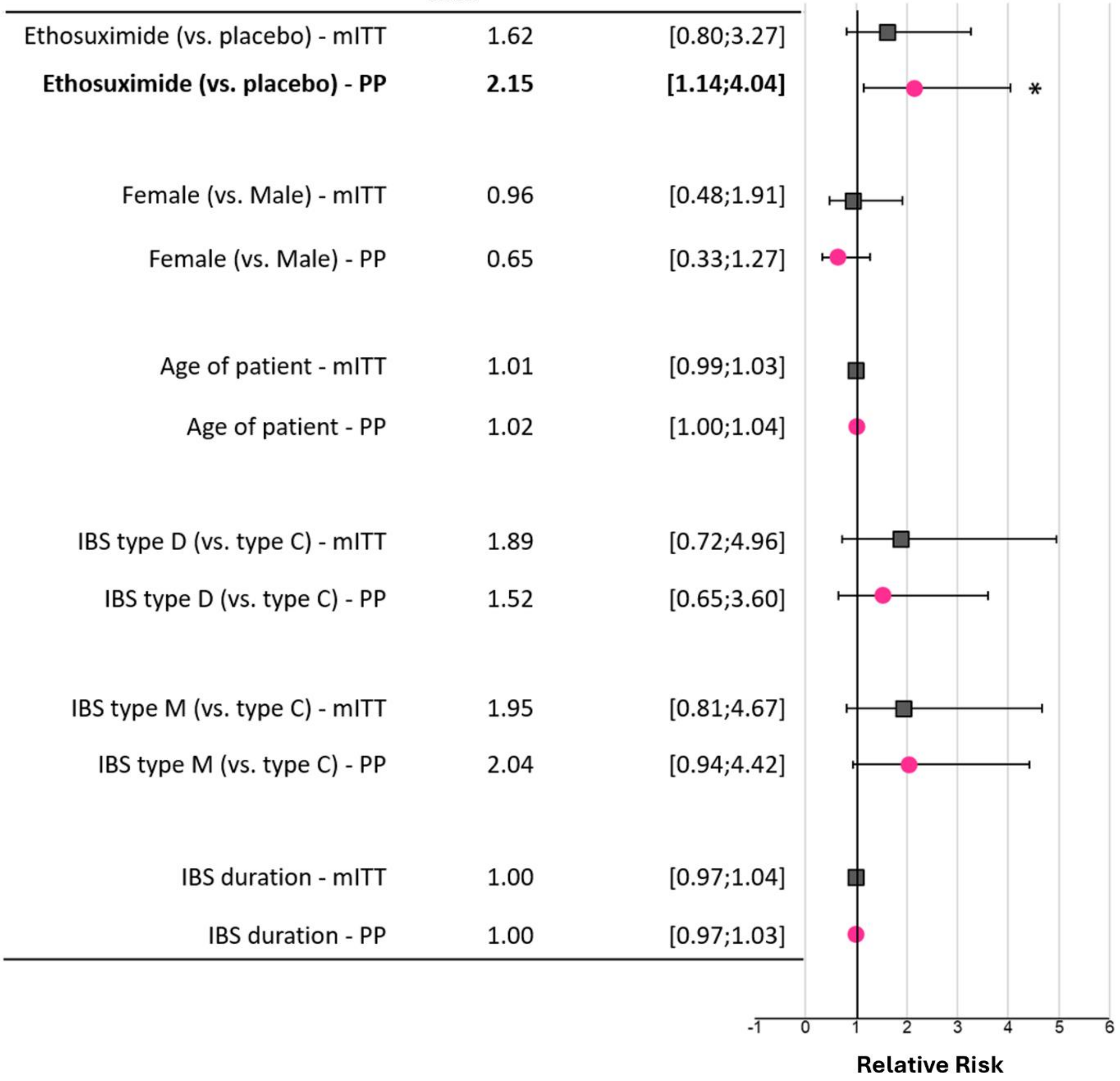

© 2026 Kerckhove N et al. *JAMA Network Open*.
