# Supplementary material for: Ethosuximide and Irritable Bowel Syndrome–Related Abdominal Pain: A Randomized Clinical Trial
Source: JAMA Netw Open. 2026 Jan 8;9(1):e2551368. doi: 10.1001/jamanetworkopen.2025.51368 (PMC12784227; doi:10.1001/jamanetworkopen.2025.51368)
Supplement: Supplement 3. — The IBSET Investigator Group (IIG) [file jamanetwopen-e2551368-s003.pdf]

Supplemental Online Content: Nonauthor Collaborators
*First name, last name, and suffix (if applicable) are required and will appear in PubMed.

***Group Name(s): IIG**

| *First Name and Middle Initial(s) | *Last Name | *Suffix (eg, Jr, III) | Academic Degrees | Institution | Location (city, state/province, country) | Role or Contribution, eg, chair, principal investigator | Group (if more than 1 Group listed in the byline) and/or Subgroup (eg, Steering Committee) |
|---|---|---|---|---|---|---|---|
| Virginie | Vernon | | MD | Université de Rouen Normandie, INSERM, ADEN UMR1073, “Nutrition, Inflammation and microbiota-gut-brain axis”, CHU Rouen, CIC-CRB 1404, service de gastroentérologie, service de physiologie | Rouen, F-76000, France | Screening and follow-up of patients | |
| Audrey | Hastier de Chelle | | MD | CHU Nice, Hôpital l'Archet 2, service de gastroentérologie | Nice, F-06202, France | Screening and follow-up of patients | |
| Cécile | Campos | | MD | CH Thiers, Service de gastroentérologie | Thiers, F-63300, France | Screening and follow-up of patients | |
| Régine | Truchi | | MD | CHU Nice, Hôpital l'Archet 2, service de gastroentérologie | Nice, F-06202, France | Screening and follow-up of patients | |
| Véronique | Vitton | | MD | Assistance Publique Hôpitaux de Marseille, Hôpital Nord, Aix Marseille Université, service de gastroentérologie | Marseille, F-13000, France | Screening and follow-up of patients | |
| Guillaume | Gourcerol | | MD | Université de Rouen Normandie, INSERM, ADEN UMR1073, “Nutrition, Inflammation and microbiota-gut-brain axis”, CHU Rouen, CIC-CRB 1404, service de gastroentérologie, service de physiologie | Rouen, F-76000, France | Screening and follow-up of patients | |
| Camille | Sautel | | MD | Université Clermont Auvergne, INSERM, UMR 1107 – NEURO-DOL, CIC 1405, CHU Clermont-Ferrand, service de gastroentérologie, service de pharmacologie | Clermont–Ferrand, F-63000, France | Screening and follow-up of patients | |

Supplemental Online Content: Nonauthor Collaborators
*First name, last name, and suffix (if applicable) are required and will appear in PubMed.

| ***First Name and Middle Initial(s)** | ***Last Name** | ***Suffix (eg, Jr, III)** | Academic Degrees | Institution | Location (city, state/province, country) | Role or Contribution, eg, chair, principal investigator | Group (if more than 1 Group listed in the byline) and/or Subgroup (eg, Steering Committee) |
|---|---|---|---|---|---|---|---|
| Pauline | Jouet | | MD | Assistance Publique Hôpitaux de Paris, Hôpital Avicenne, service d'hépato-gastroentérologie | Bobigny, F-93000, France | Screening and follow-up of patients | |
| Jamila | Lenz | | MD | Université de Rouen Normandie, INSERM, ADEN UMR1073, “Nutrition, Inflammation and microbiota-gut-brain axis”, CHU Rouen, CIC-CRB 1404, service de gastroentérologie, service de physiologie | Rouen, F-76000, France | Screening and follow-up of patients | |
| Eymeric | Chartrain | | MD | Université Clermont Auvergne, INSERM, UMR 1107 – NEURO-DOL, CIC 1405, CHU Clermont-Ferrand, service de gastroentérologie, service de pharmacologie | Clermont–Ferrand, F-63000, France | Screening and follow-up of patients | |
