# Supplementary material for: Ethosuximide and Irritable Bowel Syndrome–Related Abdominal Pain: A Randomized Clinical Trial
Source: JAMA Netw Open. 2026 Jan 8;9(1):e2551368. doi: 10.1001/jamanetworkopen.2025.51368 (PMC12784227; doi:10.1001/jamanetworkopen.2025.51368)
Supplement: Supplement 4. — Data Sharing Statement [file jamanetwopen-e2551368-s004.pdf]

Data Sharing Statement

Kerckhove. Ethosuximide and IBS-Related Abdominal Pain. *JAMA Netw Open*. Published January 08, 2026. doi:10.1001/jamanetworkopen.2025.51368

Data
Additional Information: NCT02973542, https://clinicaltrials.gov/study/NCT02973542?term=NCT02973542&rank=1
Data available: Yes
Data types: Deidentified participant data
How to access data: nkerckhove@chu-clermontferrand.fr
When available: With publication

Supporting Documents
Document types: None

Additional Information
Who can access the data: The data set was the property of the sponsor (University Hospital of Clermont-Ferrand). The principal investigator (JS) and the project manager (NK) had full access to the final data set and can provide data on written and justified request. Materials and methods are available on PubMed (PMID: 28720615).
Types of analyses: Only for IBS analyses
Mechanisms of data availability: with a signed data access agreement
